# Hepatocyte PPARα Is Essential for Triglyceride-Lowering Effect of Pemafibrate

**DOI:** 10.3390/ijms27073308

**Published:** 2026-04-06

**Authors:** Zhe Zhang, Xuguang Zhang, Chufang Qian, Pan Diao, Takero Nakajima, Takefumi Kimura, Frank J. Gonzalez, Naoki Tanaka

**Affiliations:** 1 Department of Global Medical Research Promotion, Shinshu University Graduate School of Medicine, Matsumoto 390-8621, Japan; zhangzhezz0914@163.com (Z.Z.); zhang@shinshu-u.ac.jp (X.Z.); 22hm182j@shinshu-u.ac.jp (C.Q.); 29201095@hebmu.edu.cn (P.D.); nakat@shinshu-u.ac.jp (T.N.); 2 Gastroenterology Department, University-Town Hospital of Chongqing Medical University, The Fourth Clinical College of Chongqing Medical University, Chongqing 401331, China; 3 Department of Clinical Laboratory, The Second Hospital of Hebei Medical University, Shijiazhuang 050061, China; 4 Postdoctoral Mobile Station of Clinical Medicine, Hebei Medical University, Shijiazhuang 050017, China; 5 Department of Gastroenterology, Shinshu University School of Medicine, Matsumoto 390-8621, Japan; kimuratakefumii@yahoo.co.jp; 6 Institute for Biomedical Sciences, Shinshu University, Matsumoto 390-8621, Japan; 7 Center for Cancer Research, National Cancer Institute, National Institutes of Health, Bethesda, MD 20892, USA; gonzalef@mail.nih.gov; 8 International Relations Office, Shinshu University School of Medicine, Matsumoto 390-8621, Japan

**Keywords:** pemafibrate, PPARα, hepatocyte, β-oxidation, triglycerides

## Abstract

We previously demonstrated that a clinically relevant dose of pemafibrate (PEM), a selective peroxisome proliferator-activated receptor α (PPARα) modulator (SPPARMα), reduces serum triglyceride (TG) levels in mice via hepatic PPARα activation. However, the specific contribution of hepatocyte PPARα remains unclear. To address this, male *Ppara*-floxed (*Ppara*^fl/fl^) and hepatocyte-specific *Ppara*-disrupted (*Ppara*^ΔHep^) mice were fed a diet with or without a clinically relevant dose of PEM (0.00005%) for four weeks. In *Ppara*^fl/fl^ mice, PEM significantly reduced circulating TG and non-esterified fatty acid levels by enhancing hepatic fatty acid uptake and β-oxidation. In contrast, these effects were absent in *Ppara*^ΔHep^ mice. Notably, PEM did not activate PPARα in extrahepatic tissues, including white/brown adipose tissue, kidney, and skeletal muscle in either genotype. These findings underscore the essential role of hepatocyte PPARα in mediating the pharmacological effects of PEM at clinically relevant doses.

## 1. Introduction

The liver plays a central role in regulating energy and nutrient metabolism [1,2,3]. In recent years, disruptions in energy balance and overnutrition have contributed to the increasing prevalence of metabolic syndrome, metabolic dysfunction-associated steatotic liver disease (MASLD), type 2 diabetes, and atherosclerosis [4,5,6,7]. Addressing these growing health concerns represents a major clinical challenge.

Peroxisome proliferator-activated receptors (PPARs) are nuclear receptors with three isoforms: PPARα, PPARβ/δ, and PPARγ [8]. Accumulating evidence indicates that PPARs are closely associated with the pathogenesis of diabetes, obesity, dyslipidemia, and inflammation [9,10]. Among them, PPARα serves as a key regulator of hepatic lipid homeostasis [10]. As a ligand-activated nuclear receptor, PPARα is highly expressed in hepatocytes, cardiomyocytes, proximal renal tubular cells, and brown adipocytes [10,11]. It plays a critical role in lipid transport and catabolism in the liver, contributes to glucose regulation, and is involved in both inflammation and carcinogenesis [10,11].

Fibrates are clinically used to lower serum lipid levels and are believed to act via PPARα activation [12]. However, their mechanisms of action remain incompletely understood, as clinically relevant doses of fibrates do not activate PPARα in murine models. In contrast, pemafibrate (PEM), a selective PPARα modulator (SPPARMα), has been developed as a next-generation PPARα agonist with greater selectivity and safety in humans compared to conventional fibrates [13,14]. Our previous study demonstrated that a clinically relevant dose of PEM activates hepatic PPARα and reduces serum triglyceride (TG) levels without causing hepatotoxicity in mice [15]. Nevertheless, the specific contribution of hepatocyte PPARα to these lipid-lowering effects remains unclear.

Although mouse models are widely used to investigate PPARα signaling and metabolic regulation, substantial species-specific differences exist between mice and humans. Mice exhibit hepatocellular proliferative responses and peroxisome proliferation following PPARα activation, accompanied by transcriptional regulatory programs that are largely absent in humans [8,10]. Although the findings obtained from mice experiments should be interpreted with caution, mouse models are useful to conduct mechanistic investigation and genetic manipulation.

To elucidate the role of hepatocyte PPARα in the mechanism of lipid-lowering actions of PEM, we administered a diet containing a clinically relevant dose of PEM (0.00005%) to male *Ppara*-floxed (*Ppara*^fl/fl^) and hepatocyte-specific *Ppara*-disrupted (*Ppara*^ΔHep^) mice for four weeks and evaluated their metabolic phenotypes.

## 2. Results

### 2.1. PEM Increases Hepatic PPARα Expression in Ppara^fl/fl^ Mice

To evaluate the hepatic effect of PEM, *Ppara*^fl/fl^ and *Ppara*^ΔHep^ mice were fed a 0.00005% PEM-containing diet for four weeks. The selected dietary PEM concentration (0.00005%) was based on pharmacokinetic equivalence to the clinically recommended dose in humans (0.1–0.4 mg/day). Human data showed that oral PEM administration (0.2 mg twice daily for 1 week) resulted in a maximum plasma concentration (Cmax) of 3.572 ± 1.021 ng/mL and an area under the concentration–time curve (AUC) of 12.207 ± 2.900 ng·h/mL. In mice, once-daily oral dosing at 0.075–0.1 mg/kg for 4 weeks yields comparable Cmax (2.94–3.00 ng/mL) and AUC (11.1–14.5 ng·h/mL) values. Considering average food intake (4 g/day) and body weight (~30 g), the corresponding dietary PEM concentration was estimated to be 0.000056–0.000075%. To minimize the risk of PEM accumulation and toxicity, we adopted 0.00005% as a clinically relevant dietary concentration.

**Figure 1:**
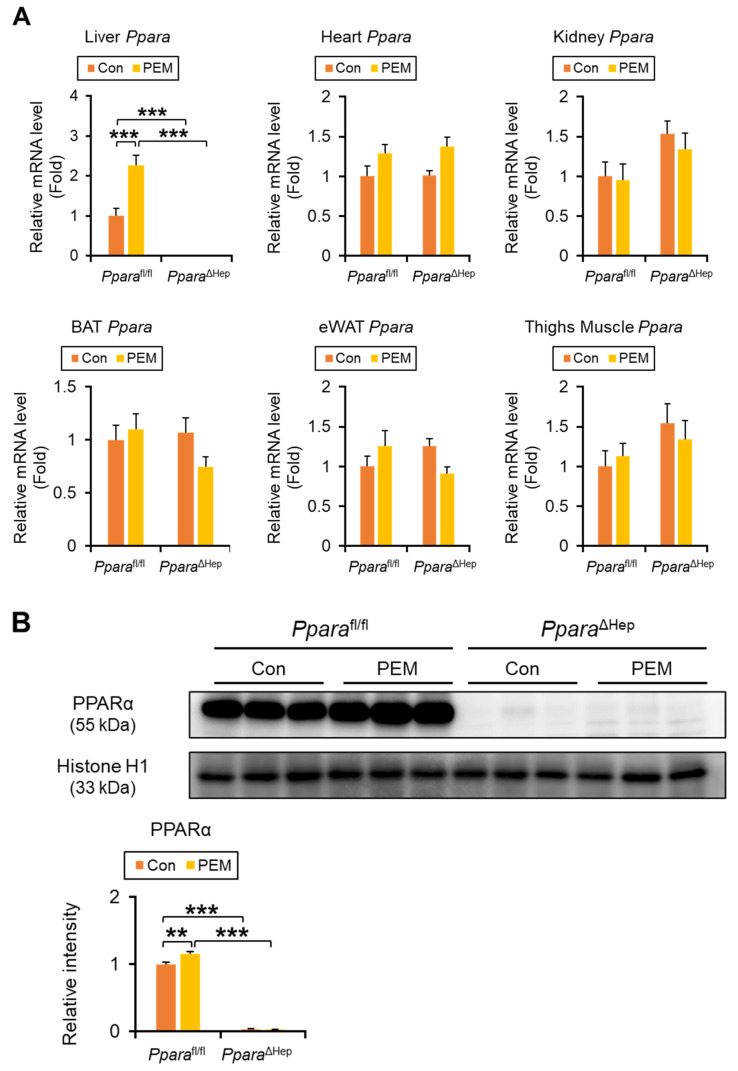
**Four-week Administration of Clinically Relevant Dose of PEM Increases Hepatic PPARα Expression in *Ppara*^fl/fl^ Mice.** (**A**) The *Ppara* mRNA levels were quantified by qPCR, normalized to that of 18s ribosomal RNA, and expressed as values relative to those of control diet-fed *Ppara*^fl/fl^ mice. (**B**) Immunoblot analysis of PPARα in the nuclear fraction of the liver. Histone H1 was used as a loading control. Band intensity was measured densitometrically, normalized to that of histone H1, and expressed as values relative to those of control diet-fed *Ppara*^fl/fl^ mice. Results were obtained from 2 independent immunoblot experiments. Data are expressed as the mean ± SEM. ** *p* < 0.01, and *** *p* < 0.001. *Ppara*^fl/fl^, *Ppara*-floxed; *Ppara*^ΔHep^, hepatocyte-specific *Ppara*-disrupted; Con, control MF diet; PEM, MF diet containing 0.00005% pemafibrate. Con n = 6–7, PEM n = 9–10 in each genotype.

*Ppara* mRNA was undetectable in the liver of *Ppara*^ΔHep^ mice using quantitative polymerase chain reaction (qPCR), while expression levels in the heart, kidney, brown adipose tissue (BAT), epididymal white adipose tissue (eWAT), and thigh skeletal muscle were comparable between genotypes, confirming liver-specific *Ppara* gene disruption (Figure 1A). Two-way ANOVA revealed a significant genotype × PEM interaction for hepatic *Ppara* expression, and simple effects analysis showed that PEM treatment significantly increased *Ppara* mRNA levels and nuclear PPARα protein expression in *Ppara*^fl/fl^ mice, whereas no significant changes were observed in *Ppara*^ΔHep^ mice (Figure 1A,B). In eWAT, *Ppara* expression also exhibited a significant genotype × PEM interaction, although post hoc pairwise comparisons did not reach significance, with PEM tending to increase expression preferentially in *Ppara*^fl/fl^ mice (Figure 1A,B).

**Figure 2:**
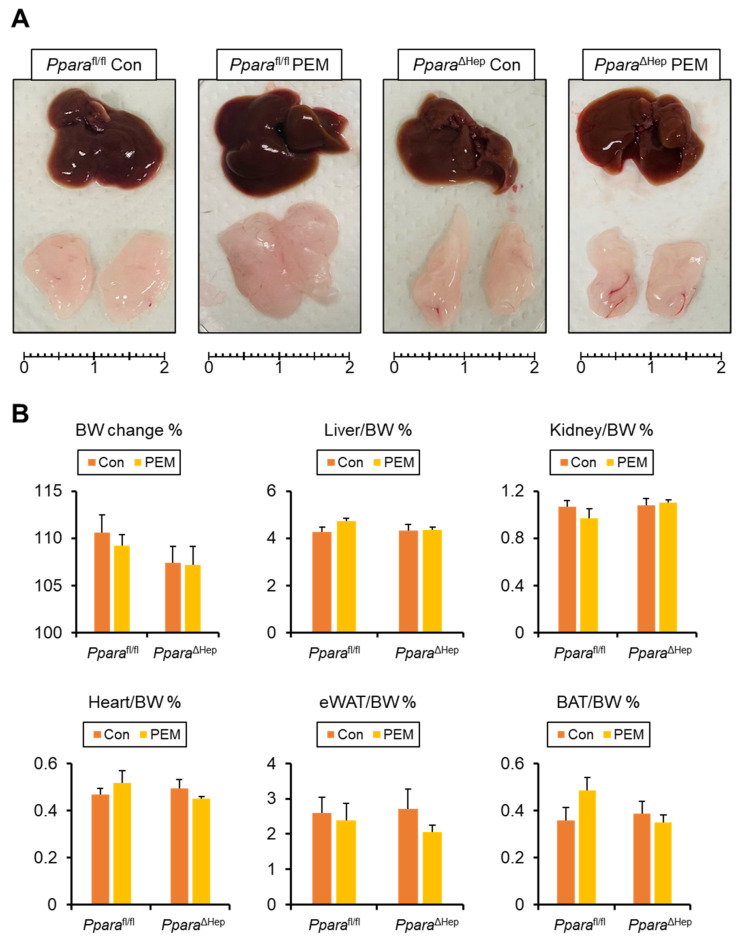
**Gross Appearance of Mice Treated with Clinically Relevant Dose of PEM for 4 Weeks.** (**A**) Gross appearance of the liver and epididymal white adipose tissue (eWAT). Scale bar = 2 cm. (**B**) Body weight (BW) change and the ratio of liver, kidney, heart, eWAT, and subcapsular brown adipose tissue (BAT) weight to BW. Data are expressed as the mean ± SEM. *Ppara*^fl/fl^, *Ppara*-floxed; *Ppara*^ΔHep^, hepatocyte-specific *Ppara*-disrupted; Con, control MF diet; PEM, MF diet containing 0.00005% pemafibrate. Con n = 6–7, PEM n = 9–10 in each genotype.

All mice remained healthy and maintained similar food intake throughout the study. Body weight changes at the end point or the liver, kidney, heart, eWAT, and BAT-to-body weight ratio did not significantly differ among groups (Figure 2A,B). No significant phenotypic differences were observed between genotypes under control diet conditions.

### 2.2. PEM Reduces Serum TG and NEFA in a Hepatocyte PPARα-Dependent Manner

**Figure 3:**
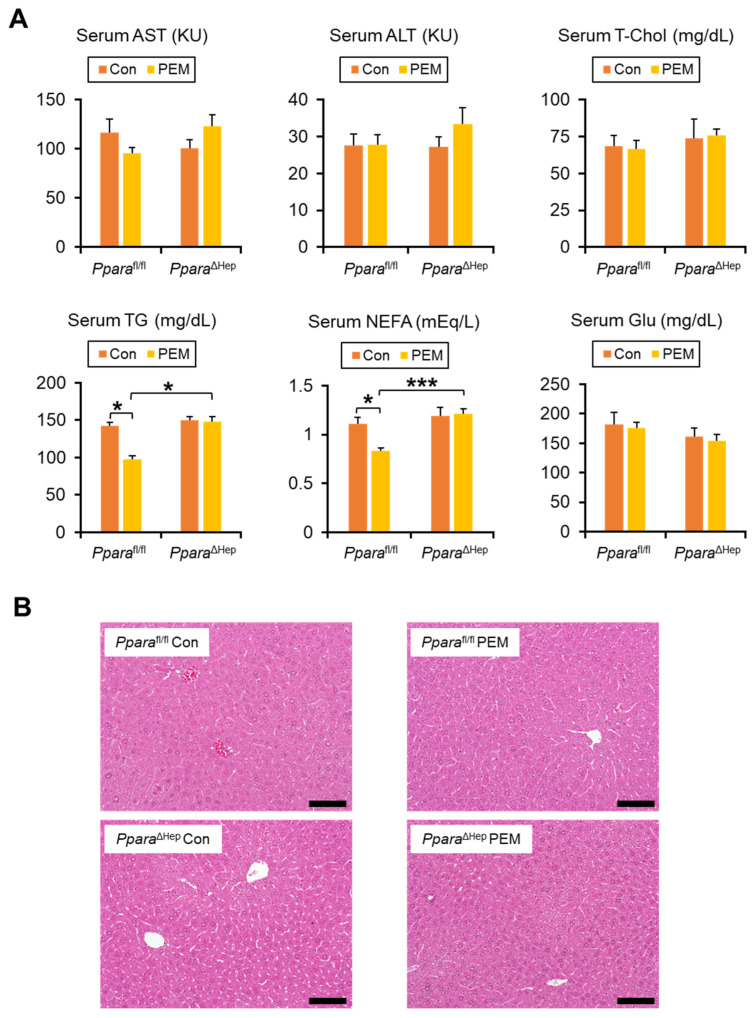
**Clinically Relevant Dose of PEM Reduces Serum TG and NEFA in *Ppara*^fl/fl^ Mice, but Not *Ppara*^ΔHep^ Mice.** (**A**) Serum aspartate aminotransferase (AST) and alanine aminotransferase (ALT) activities and serum total cholesterol (T-Chol), triglyceride (TG), non-esterified fatty acid (NEFA), and glucose (Glu) levels. Data are expressed as the mean ± SEM. * *p* < 0.05, and *** *p* < 0.001. (**B**) Representative photomicrographs of hematoxylin and eosin-stained liver sections. Scale bar = 50 μm. *Ppara*^fl/fl^, *Ppara*-floxed; *Ppara*^ΔHep^, hepatocyte-specific *Ppara*-disrupted; Con, control MF diet; PEM, MF diet containing 0.00005% pemafibrate. Con n = 6–7, PEM n = 9–10 in each genotype.

After PEM treatment, serum levels of aspartate aminotransferase (AST), alanine aminotransferase (ALT), total cholesterol (T-Chol), glucose (Glu), and total bile acids (TBA) were unchanged, while serum phospholipid (PL) levels decreased in *Ppara*^ΔHep^ mice (Figure 3A, Supplementary Figure S1A). As expected, genotype and PEM treatment interacted significantly with serum TG and non-esterified fatty acids (NEFA) levels. PEM significantly lowered serum TG and NEFA concentrations in *Ppara*^fl/fl^ mice, whereas this effect was absent in *Ppara*^ΔHep^ mice (Figure 3A). Hepatic contents of T-Chol, TG, NEFA, and PL, as well as liver histology, showed no significant differences between groups (Figure 3B, Supplementary Figure S1B).

### 2.3. PEM Induces Fatty Acid β-Oxidation Genes in a Hepatocyte PPARα-Dependent Manner

**Figure 4:**
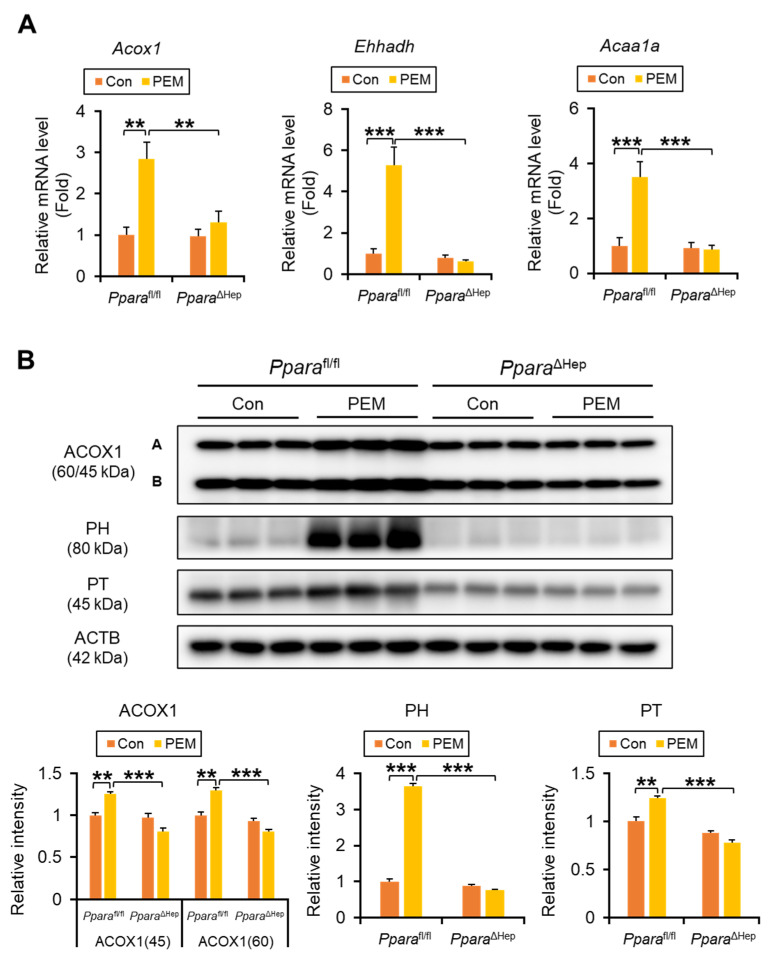
**Clinically Relevant Dose of PEM Enhances Peroxisomal β-Oxidation Enzymes in *Ppara*^fl/fl^ Mice, but Not *Ppara*^ΔHep^ Mice.** (**A**) The mRNA levels of genes encoding peroxisomal β-oxidation enzymes (*Acox1*, *Ehhadh*, and *Acaa1a*) were quantified by qPCR, normalized to that of 18s ribosomal RNA, and expressed as values relative to those of control diet-fed *Ppara*^fl/fl^ mice. (**B**) Immunoblot analysis of peroxisomal acyl-coenzyme A oxidase 1 (ACOX1), peroxisomal hydratase (PH) and peroxisomal thiolase (PT) in the liver. The β-actin (ACTB) was used as a loading control. Band intensity was measured densitometrically, normalized to that of ACTB, and expressed as values relative to those of control diet-fed *Ppara*^fl/fl^ mice. Results were obtained from 2 independent immunoblot experiments. Data are expressed as the mean ± SEM. ** *p* < 0.01, and *** *p* < 0.001. *Ppara*^fl/fl^, *Ppara*-floxed; *Ppara*^ΔHep^, hepatocyte-specific *Ppara*-disrupted; Con, control MF diet; PEM, MF diet containing 0.00005% pemafibrate. Con n = 6–7, PEM n = 9–10 in each genotype.

We next examined the transcriptional regulation of genes involved in fatty acid (FA) β-oxidation. Significant genotype × PEM interactions were identified for hepatic expression of key peroxisomal FA β-oxidation enzymes, such as *Acox1* (acyl-coenzyme A [CoA] oxidase 1, ACOX1), *Ehhadh* (enoyl-CoA hydratase and 3-hydroxyacyl CoA dehydrogenase), and *Acaa1a* (peroxisomal thiolase, PT). In *Ppara*^fl/fl^ mice, PEM markedly increased hepatic *Acox1*, *Ehhadh*, and *Acaa1a* mRNA (Figure 4A). These changes were not observed in PEM-treated *Ppara*^ΔHep^ mice and were confirmed by immunoblotting of peroxisomal FA β-oxidation enzymes, including ACOX1, peroxisomal hydratase (PH), and PT (Figure 4B).

**Figure 5:**
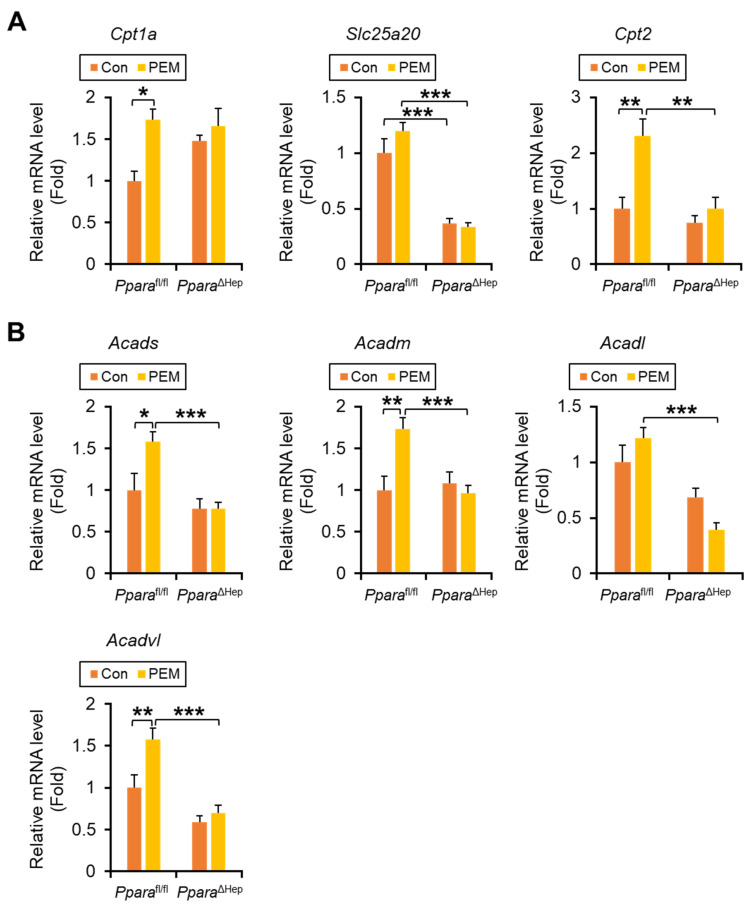

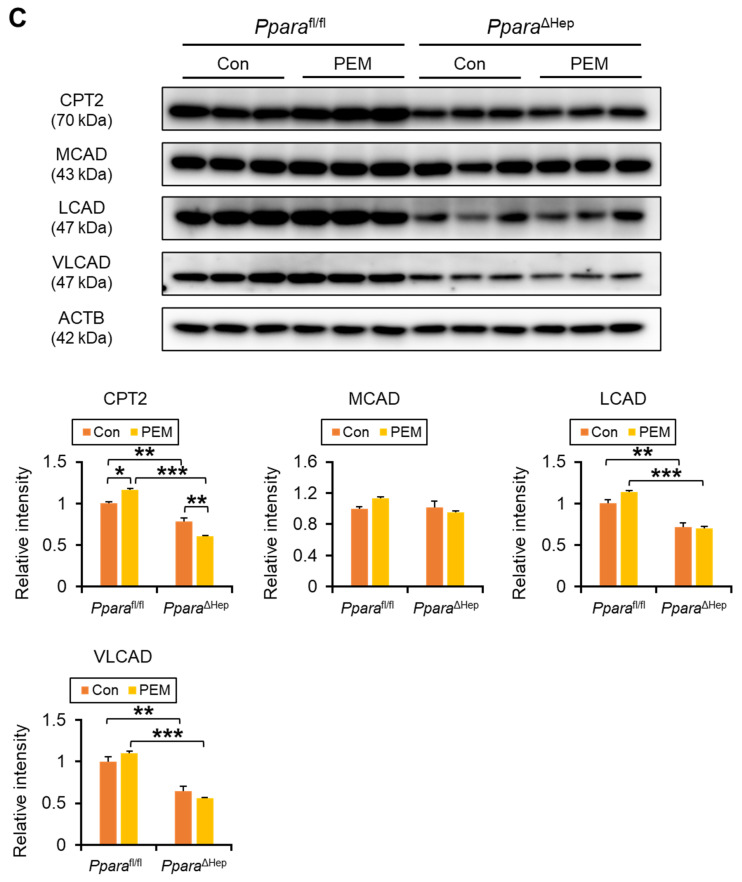
**Clinically Relevant Dose of PEM Enhances FA Entry into Mitochondria and Ensuing β-Oxidation in *Ppara*^fl/fl^ Mice, but Not *Ppara*^ΔHep^ Mice.** (**A**,**B**) The mRNA levels of genes involved in FA shuttling into mitochondria (**A**) (*Cpt1a*, *Slc25a20*, and *Cpt2*) and mitochondrial β-oxidation (**B**) (*Acads*, *Acadm*, *Acadl*, and *Acadvl*) were quantified by qPCR, normalized to that of 18s ribosomal RNA, and expressed as values relative to those of control diet-fed *Ppara*^fl/fl^ mice. (**C**) Immunoblot analysis of CPT2, MCAD, LCAD, and VLCAD in the liver. The β-actin (ACTB) was used as a loading control. Band intensity was measured densitometrically, normalized to that of ACTB, and expressed as values relative to those of control diet-fed *Ppara*^fl/fl^ mice. Results were obtained from 2 independent immunoblot experiments. Data are expressed as the mean ± SEM and the same samples used as Figure 4A,B were adopted in the respective experiment. * *p* < 0.05, ** *p* < 0.01, and *** *p* < 0.001. *Ppara*^fl/fl^, *Ppara*-floxed; *Ppara*^ΔHep^, hepatocyte-specific *Ppara*-disrupted; Con, control MF diet; PEM, MF diet containing 0.00005% pemafibrate. Con n = 6–7, PEM n = 9–10 in each genotype.

Significant genotype × PEM interactions were also observed for mitochondrial FA β-oxidation genes, including *Cpt2* (carnitine palmitoyl-CoA transferase 2, CPT2), *Acads* (short-chain acyl-CoA dehydrogenase, SCAD), *Acadm* (medium-chain acyl-CoA dehydrogenase, MCAD), and *Acadl* (long-chain acyl-CoA dehydrogenase, LCAD), with PEM increasing their expression exclusively in *Ppara*^fl/fl^ mice. For *Cpt1a* (carnitine palmitoyl-CoA transferase 1a) and *Acadvl* (very-long-chain acyl-CoA dehydrogenase, VLCAD), the interaction terms did not reach statistical significance (*p* = 0.09 and 0.06, respectively), although PEM tended to induce higher expression preferentially in *Ppara*^fl/fl^ mice. In contrast, *Slc25a20* mRNA (solute carrier family 25 member 20, also named as carnitine-acylcarnitine translocase) showed a robust main effect of genotype (*p* < 0.001), indicating that hepatic *Ppara* deficiency markedly reduced its expression regardless of PEM treatment (Figure 5A,B).

Immunoblot analysis revealed significant genotype × PEM interactions for CPT2 and MCAD protein expression. PEM treatment increased CPT2 levels in PEM-treated *Ppara*^fl/fl^ mice, but not in *Ppara*^ΔHep^ mice. For MCAD, although the interaction term was significant (*p* = 0.03), posthoc simple effect comparisons did not reach significance. LCAD and VLCAD showed interaction effects that did not reach statistical significance (both *p* = 0.06), with PEM exhibiting a tendency to increase their expression preferentially in *Ppara*^fl/fl^ mice (Figure 5C). These data support the role of hepatic PPARα activation in promoting FA β-oxidation in both peroxisomes and mitochondria, thereby contributing to serum TG reduction.

### 2.4. PEM Enhances Hepatic FA Uptake and Activation in a Hepatocyte PPARα-Dependent Manner

**Figure 6:**
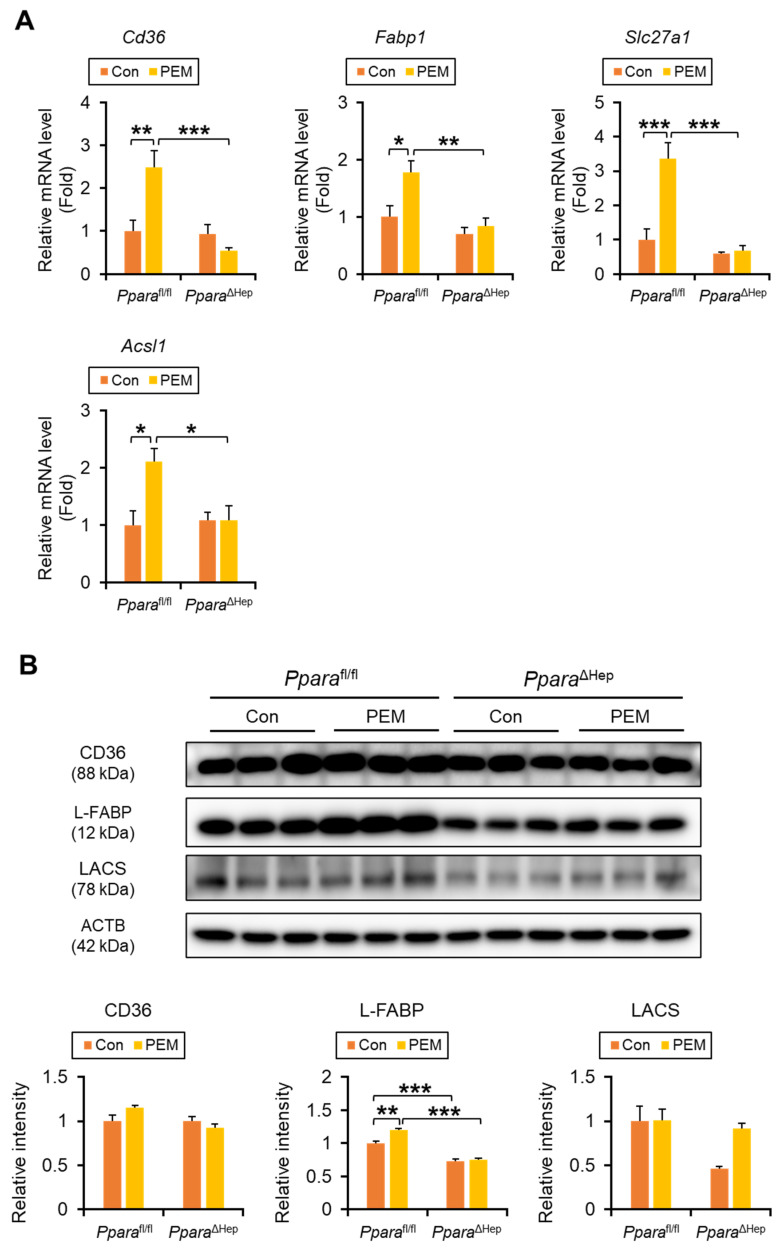
**Clinically Relevant Dose of PEM Enhances FA Uptake into the Liver in *Ppara*^fl/fl^ Mice, but Not *Ppara*^ΔHep^ Mice.** (**A**) The mRNA levels of genes encoding *Cd36*, *Fabp1*, *Slc27a1*, and *Acsl1* were quantified by qPCR, normalized to that of 18s ribosomal RNA, and expressed as values relative to those of control diet-fed *Ppara*^fl/fl^ mice. (**B**) Immunoblot analysis of CD36, L-FABP, and LACS in the liver. The β-actin (ACTB) was used as a loading control. Band intensity was measured densitometrically, normalized to that of ACTB, and expressed as values relative to those of control diet-fed *Ppara*^fl/fl^ mice. Results were obtained from 2 independent immunoblot experiments. Data are expressed as the mean ± SEM and the same samples used as Figure 4A,B were adopted in the respective experiment. * *p* < 0.05, ** *p* < 0.01, and *** *p* < 0.001. *Ppara*^fl/fl^, *Ppara*-floxed; *Ppara*^ΔHep^, hepatocyte-specific *Ppara*-disrupted; Con, control MF diet; PEM, MF diet containing 0.00005% pemafibrate. Con n = 6–7, PEM n = 9–10 in each genotype.

To further elucidate the mechanism of PEM action, we analyzed the expression of FA uptake and transport genes. Significant genotype × PEM interactions were observed for *Cd36* (CD36, also named as FA translocase), *Slc27a1* (FA transport protein 1), and *Acsl1* (long-chain acyl-CoA synthetase, LACS), with PEM increasing their hepatic mRNA levels in *Ppara*^fl/fl^ mice, but not in *Ppara*^ΔHep^ mice. For *Fabp1* (liver FA-binding protein, L-FABP), although the interaction was not statistically significant (*p* = 0.07), pairwise comparisons revealed that PEM significantly increased *Fabp1* expression in *Ppara*^fl/fl^ mice. (Figure 6A). Immunoblot analysis confirmed significant genotype × PEM interactions for CD36 and L-FABP, although groupwise comparisons for CD36 were not significant (Figure 6B).

### 2.5. PEM Upregulates Hepatic MTP and FGF21 via PPARα in Hepatocytes

**Figure 7:**
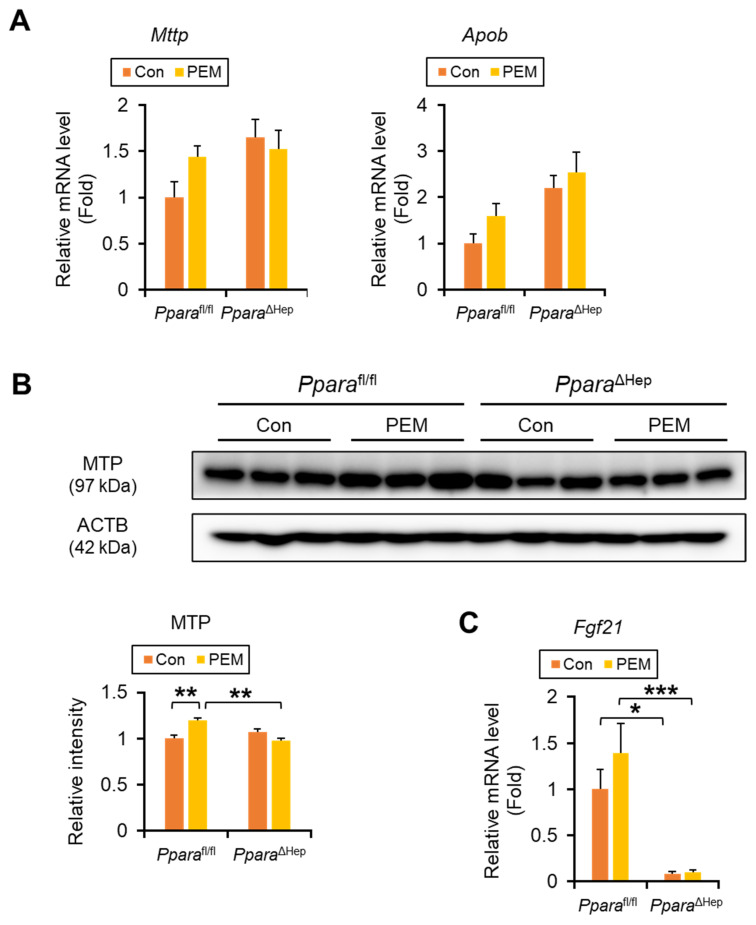
**Clinically Relevant Dose of PEM Enhances MTP Expression in *Ppara*^fl/fl^ Mice, but Not *Ppara*^ΔHep^ Mice.** (**A**) The mRNA levels of genes encoding *Mttp* and *Apob*, responsible for VLDL secretion from the liver, were quantified by qPCR, normalized to that of 18s ribosomal RNA, and expressed as values relative to those of control diet-fed *Ppara*^fl/fl^ mice. (**B**) Immunoblot analysis of MTP in the liver. The β-actin (ACTB) was used as a loading control. Band intensity was measured densitometrically, normalized to that of ACTB, and expressed as values relative to those of control diet-fed *Ppara*^fl/fl^ mice. Results were obtained from 2 independent immunoblot experiments. (**C**) The mRNA levels of genes encoding *Fgf21* in the liver. Data are expressed as the mean ± SEM and the same samples used as Figure 4A,B were adopted in the respective experiment. * *p* < 0.05, ** *p* < 0.01, and *** *p* < 0.001. *Ppara*^fl/fl^, *Ppara*-floxed; *Ppara*^ΔHep^, hepatocyte-specific *Ppara*-disrupted; Con, control MF diet; PEM, MF diet containing 0.00005% pemafibrate. Con n = 6–7, PEM n = 9–10 in each genotype.

PEM treatment did not affect the expression of genes involved in *de novo* FA synthesis (*Fasn* and *Acaca*), TG synthesis (*Dgat1/2*), or TG hydrolysis (adipose TG lipase [*Pnpla2*] and hepatic lipase [*Lipc*]), in either genotype (Supplementary Figure S2). Although *Mttp* (microsomal TG transfer protein, MTP) and *Apob* (apolipoprotein B) mRNA levels were unchanged by PEM treatment (Figure 7A), immunoblotting revealed a significant genotype × PEM interaction with increasing MTP protein levels in PEM-treated *Ppara*^fl/fl^ mice (Figure 7B). For *Fgf21* (fibroblast growth factor 21, FGF21) mRNA, the genotype × PEM interaction was not significant, but PEM-induced *Fgf21* expression tended to increase only in *Ppara*^fl/fl^ mice (Figure 7C). These results suggest that PEM may facilitate very low-density lipoprotein (VLDL) assembly/secretion and increase FGF21 levels through hepatic PPARα activation.

### 2.6. Minimal Activation of PPARα in Extrahepatic Tissues by PEM

To assess potential off-target effects, we examined PPARα activation in extrahepatic tissues. Despite the increase in hepatic PPARα target genes (e.g., *Acox1*, *Ehhadh*, *Acadvl*, *Fabp1*, and *Acsl1*) in PEM-treated *Ppara*^fl/fl^ mice (Figure 4, Figure 5 and Figure 6), histological analysis and gene expression profiling revealed minimal effects of PEM in the heart, kidney, BAT, and skeletal muscle (Supplementary Figures S3–6). In eWAT, similar to the pattern observed for *Ppara*, *Acox1*, *Acaa1a*, *Acadm*, and *Acadl* also exhibited significant genotype × PEM interactions, but post hoc pairwise comparisons did not reach significance (Supplementary Figure S7).

### 2.7. Minimal Impact of PEM on WAT Lipolysis, Adipogenesis, and Browning

To investigate whether PEM alters white adipose tissue metabolism, we examined the mRNA expression of genes involved in lipolysis (e.g., *Pnpla2*, carboxylesterase 1d [*Ces1d*], and hormone-sensitive lipase [*Lipe*]), adipogenesis (perilipin 1 [*Plin1*] and fat-specific protein 27 [*Cidec*]), browning (type 2 deiodinase [*Dio2*] and uncoupling protein 1 [*Ucp1*]), and adipokine synthesis (adiponectin [*Adipoq*]). Although *Pnpla2*, *Lipe*, *Plin1*, and *Adipoq* also exhibited significant genotype × PEM interactions, no significant differences were observed between individual groups (Supplementary Figure S8), suggesting that while PEM influences the expression of these adipose genes in a liver PPARα-dependent manner, they are unlikely to be the primary mediators of PEM-induced reductions in serum TG levels and clinically relevant dose of PEM has little effect on eWAT remodeling under the conditions tested.

### 2.8. Minimal Effect of PEM on Peroxisome Proliferation and Oxidative Stress

We determined whether the administered dose induced peroxisome proliferation or hepatic oxidative stress. Western blot analysis of the peroxisomal membrane protein PMP70 and the peroxisomal matrix enzyme catalase revealed no significant differences between treated and control groups, indicating the absence of peroxisomal compartment expansion (Supplementary Figure S9). Hepatic oxidative stress was further assessed by measuring lipid peroxidation and antioxidant stress–response signaling. Hepatic thiobarbituric acid reactive substances (TBARS) levels were comparable between the groups, and the mRNA expression levels of key oxidative stress–responsive genes, including *Nrf2* (nuclear factor erythroid derived 2 like 2, called as nuclear factor erythroid 2-related factor 2), *Sod1* (superoxide dismutase 1), *Sod2, Hmox1* (heme oxygenase 1), and *Nqo1* (NAD(P)H quinone dehydrogenase 1), were not induced by PEM treatment (Supplementary Figure S10). These data demonstrate that clinically relevant dose of PEM does not induce hepatic oxidative stress.

### 2.9. Minimal Effect of PEM on Activation of Other Xenobiotic Nuclear Receptors

To exclude the possibility that PEM acts as a xenobiotic and activates classical xenobiotic-sensing nuclear receptors other than PPARα, we examined the hepatic expression of established target genes of aryl hydrocarbon receptor (AhR), pregnane X receptor (PXR), and constitutive androstane receptor (CAR). The mRNA levels of AhR target genes (*Cyp1a1*, *Cyp1a2*, and *Cyp1b1*), PXR target genes (*Cyp3a11* and *Cyp3a25*), and CAR target genes (*Cyp2b10* and *Cyp2c55*) were not significantly altered in PEM-treated mice compared with controls (Supplementary Figure S11). Two-way ANOVA revealed no significant effects of genotype for most of these genes, except for the CAR target gene *Cyp2c55* in the PEM-treated group (Supplementary Figure S11). These results indicate that PEM does not activate AhR, PXR, or CAR signaling pathways under the present experimental condition.

## 3. Discussion

This study clearly demonstrated that PEM could reduce serum TG/NEFA in a hepatocyte PPARα-dependent manner, likely due to activating PPARα in the hepatocytes and enhancing FA uptake and β-oxidation. Indeed, the lipid-lowering effect of PEM was not detected by hepatocyte-specific PPARα disruption in mice. In this experiment using clinically relevant dose of PEM, PPARα in the kidneys, adipose tissue, or thigh muscle tissue were not activated. Our results provide key insights regarding the crucial role of hepatocyte PPARα on the pharmacological action of PEM.

When evaluating the mechanism of action of clinically used drugs in animal models, it is essential to replicate human pharmacokinetic parameters such as Cmax and AUC as closely as possible. In our study, we administered 0.00005% PEM mixed in the standard diet, which corresponds to approximately 0.075–0.1 mg/day—doses reported to achieve human-equivalent plasma exposure levels [15]. Under these conditions, PEM significantly activated PPARα and reduced serum TG and NEFA levels in mice in a PPARα-dependent manner without elevating serum AST or ALT, a profile consistent with observations in human clinical setting [16,17,18]. Additionally, no peroxisome proliferation or increased hepatic oxidative stress were detected in mice with the clinically relevant dose of PEM, indicating efficient and safe PPARα activation in this setting. In contrast, previous studies using a higher dietary concentration of PEM (0.00033%) demonstrated robust hepatic PPARα activation but also reported marked ALT elevation [19]. Therefore, the present study likely provides a more faithful representation of the clinical effects of PEM.

We further confirmed that hepatocyte PPARα plays a central role in the lipid-lowering effect of clinically relevant PEM dosing. Given that PEM is predominantly accumulated in the liver and excreted via bile [20,21], it is pharmacologically reasonable that hepatic PPARα is the primary molecular target. In contrast, PEM did not induce PPARα activation in extrahepatic organs such as kidney, adipose tissue, or skeletal muscle, suggesting a liver-centric mode of action. Interestingly, in the present study, weak induction of ACOX1 and MCAD was also observed in the hearts of both *Ppara*^fl/fl^ and *Ppara*^ΔHep^ mice. While this might reflect minimal off-target effects or systemic feedback mechanisms, the absence of serum lipid reduction in knockout mice clearly indicates that such extrahepatic activation, if any, plays only a negligible role in the overall pharmacological effect.

The distinction between hepatic and extrahepatic responses to PEM is particularly important when considering long-term administration. Although our current study involved a 4-week administration of PEM, in clinical practice, patients often receive the drug for several months to a year or more. Therefore, it remains to be determined whether longer-term treatment could lead to beneficial metabolic effects in extrahepatic tissues via hepatocyte-derived mediators [4,8,10,22]. Moreover, it is currently unclear whether such beneficial effects—if observed—are the result of direct pharmacological effects of PEM in those tissues, or whether they occur indirectly as a downstream consequence of hepatic PPARα activation. For example, the elevated FGF21 levels observed in this study may act as an endocrine mediator, influencing metabolic activity in distant tissues such as adipose or cardiac muscle [23]. Disentangling the direct versus indirect pathways of PEM’s systemic actions will be crucial for understanding its full therapeutic potential and safety in long-term clinical use.

Although MTP is not a direct target gene of PPARα, PEM administration was associated with increased hepatic MTP expression, consistent with our previous findings [15]. This observation is intriguing in light of reports showing an inverse correlation between MTP expression and MASLD severity [24,25,26]. In addition to promoting β-oxidation, PEM-induced upregulation of MTP may also contribute to its hepatoprotective effects in MASLD, as recently suggested in clinical studies [27,28,29]. However, increased MTP expression is directly linked to enhanced VLDL assembly and secretion, which could theoretically increase circulating TG levels [30,31]. In our study, PEM treatment did not cause a significant elevation in plasma TG, indicating that the increase in hepatic lipid export did not translate into systemic hypertriglyceridemia or dyslipidemia. 

The induction of PPARα itself and *Dgat2* observed in our study raises the possibility that PEM may influence additional nuclear receptor pathways beyond direct modulation of PPARα signaling. Because many xenobiotics activated hepatic metabolic programs through classical xenobiotic-sensing nuclear receptors, it was important to determine whether PEM might function as a xenobiotic ligand [8]. To address this possibility, we examined the hepatic expression of well-established target genes of the major xenobiotic sensors AhR, CAR, and PXR. However, the expression levels of representative target genes were not significantly altered under our experimental conditions. These findings indicate that PEM does not activate canonical xenobiotic-responsive pathways mediated by AhR, CAR, or PXR at the administered dose. We noted increased *Cyp2c55* expression in PEM-treated *Ppara*^ΔHep^ mice compared with similarly treated *Ppara*^fl/fl^ mice. Although the precise mechanism remains unclear, hepatocyte *Ppara* disruption might affect the signaling pathway of other nuclear receptors under the special situation.

Furthermore, while some reports have suggested lipolytic effects of PEM on adipose tissue [32], our gene expression analyses found no significant changes in key regulators of lipolysis. Hepatic FGF21 acts on adipose tissue and peripheral organs to promote FA oxidation, enhance energy expenditure, and improve lipid utilization [22,33,34]. Previous studies have shown that FGF21 increases TG clearance by enhancing lipoprotein lipase activity and modulating VLDL metabolism and promoting lipid oxidation in extrahepatic tissues [35]. Although PEM treatment showed a non-significant upward trend in hepatic FGF21 expression, we did not observe significant transcriptional changes in key lipid metabolic regulators in adipose tissue. This suggests that the adipose effects of PEM may occur through indirect mechanisms rather than direct transcriptional regulation within adipose tissue.

By utilizing hepatocyte-specific *Ppara*-disrupted mice, we have clarified the essential contribution of hepatic PPARα to the pharmacological action of PEM. This model is particularly valuable for dissecting the hepatic versus systemic effects of PEM, especially in tissues such as the liver, heart, and kidney where PPARα is abundantly expressed. Applying this clinically relevant PEM dosing regimen to disease models will enable comprehensive investigation into the molecular mechanisms underlying both its metabolic and organ-protective actions. Our findings thus offer important implications for understanding the therapeutic potential of PEM preferentially targeting liver and systemic metabolic disorders.

## 4. Methods

### 4.1. Mice

*Ppara*^fl/fl^ mice on a C57BL/6N background were generated as previously described [36]. To produce *Ppara*^fl/fl^ mice possessing Cre recombinase gene (*Ppara*^ΔHep^), *Ppara*^fl/fl^ mice were crossed with C57BL/6N transgenic mice expressing Cre recombinase under the control of the albumin promoter (The Jackson Laboratory, Boston, MA, USA) and male *Ppara*^fl/wild type^ littermates having Cre recombinase gene were obtained as a F1 generation. Next, male *Ppara*^fl/wild type^ littermates having Cre recombinase gene were mated with female *Ppara*^fl/fl^ mice, and male *Ppara*^ΔHep^ mice were obtained. Accordingly, one male *Ppara*^ΔHep^ mice and two female *Ppara*^fl/fl^ mice were housed in each breeding cage to obtain male *Ppara*^fl/fl^ or *Ppara*^ΔHep^ mice. These mice were used for this PEM experiment. Genotypes were confirmed by PCR-based genotyping. Mice were maintained under controlled conditions (25 °C, 12-h light/dark cycle, constant humidity) with free access to water and standard laboratory chow until the start of the intervention.

### 4.2. PEM Administration

All experimental procedures were approved by the Animal Care and Use Committee of Shinshu University School of Medicine (ID: 020090). PEM was provided from Kowa Company, Ltd. (Tokyo, Japan). Mice were fed either a control MF diet or an MF diet containing 0.00005% PEM for 4 weeks. The detailed composition of the MF diet is provided in Supplementary Table S1. Both diets were purchased from Oriental Yeast Co., Ltd. (Tokyo, Japan) and were isocaloric.

Twelve-week-old male *Ppara*^fl/fl^ and *Ppara*^ΔHep^ mice (25–35 g) were randomly assigned to control or PEM groups (n = 6–10 per group). After 4 weeks of diet intervention, mice were fasted for 6 h, anesthetized with CO_2_, and euthanized. Blood was collected and centrifuged twice to obtain serum. Tissues were excised, weighed, and divided: one portion was fixed in 10% neutral-buffered formalin for histological analysis, and the remainder was snap-frozen in dry ice and stored at −80 °C for further assays.

### 4.3. Histological Analysis

Samples of liver, kidney, heart, skeletal muscle, BAT, and eWAT were fixed in 10% neutral-buffered formalin and processed for paraffin embedding. Tissue sections (4 µm) were stained with hematoxylin and eosin method and examined under a light microscope using a ×20 objective lens (final magnification ×200). For each tissue type, at least three non-overlapping regions per mouse were evaluated [37,38,39,40].

### 4.4. Biochemical Measurements

Serum concentrations of AST, ALT, T-Chol, TG, NEFA, PL, Glu, and TBA were measured using commercial enzymatic kits (Wako Pure Chemical Industries, Ltd., Osaka, Japan) [37,40]. Lipids in liver tissue were extracted using a hexane:isopropanol mixture and quantified with the same enzymatic assays, following established protocols [37,40]. Hepatic levels of lipid peroxides were measured as TBARS using OxiSelect™ TBARS assay kit (Cell Biolabs, Inc., San Diego, CA, USA).

### 4.5. Gene Expression Analysis

For RNA isolation, ~30 mg of liver, kidney, or BAT was homogenized, whereas heart and skeletal muscle were disrupted by sonication. Approximately 100 mg of eWAT was homogenized in 0.5 mL of TRI Reagent (Molecular Research Center, Inc., Cincinnati, OH, USA). Total RNA was extracted using the NucleoSpin RNA Plus kit (MACHEREY-NAGEL GmbH & Co. KG, Düren, Germany) or according to the manufacturer’s instructions for TRI Reagent. Complementary DNA (cDNA) synthesis was carried out using oligo-dT and random primers with the PrimeScript RT Reagent Kit (Perfect Real Time; Takara Bio Inc., Shiga, Japan). Quantitative PCR (qPCR) was performed using SYBR Green Master Mix (Toyobo Co., Ltd., Osaka, Japan) on a QuantStudio 3 Real-Time PCR System (Thermo Fisher Scientific, Waltham, MA, USA). Transcript levels were normalized to 18S rRNA and expressed as fold change relative to those of *Ppara*^fl/fl^ mice fed the control diet [37,40]. Primer sequences are provided in Supplementary Table S2.

### 4.6. Immunoblotting

Approximately 40 mg of liver tissue was lysed in buffer containing 0.25 M sucrose, 25 mM Tris-HCl, 25 mM KCl, 5 mM MgCl_2_, 0.5% Triton X-100, and 1 mM DTT (pH 7.4), supplemented with protease and phosphatase inhibitors (Thermo Fisher Scientific, 1:100 dilution). For nuclear protein extraction, ~50 mg of liver tissue was homogenized on ice in a chilled Dounce homogenizer (Wheaton, NJ, USA) and processed using the NE-PER^®^ Nuclear and Cytoplasmic Extraction Kit (Thermo Fisher Scientific). Protein concentrations were determined by the BCA assay (Pierce, IL, USA).

Whole liver lysates (40–60 µg) and nuclear extracts (40 µg) were separated by sodium dodecyl sulfate-polyacrylamide gel electrophoresis (7.5–15% or 12.5%, depending on target protein size) and transferred to PVDF or nitrocellulose membranes (Merck Millipore Ltd., Darmstadt, Germany). Membranes were blocked with 3–10% skim milk in Tris buffer and incubated overnight at 4 °C with primary antibodies (Supplementary Table S3). After washing in TBST, membranes were incubated with secondary antibodies conjugated to alkaline phosphatase or horseradish peroxidase (Jackson ImmunoResearch, 1:4000) and visualized using NBT/BCIP (Pierce) or chemiluminescence.

Protein bands were detected using the ChemiDoc Touch Imaging System (Bio-Rad, Hercules, CA, USA) and identified based on molecular weight markers (PM2500, Smobio, Hsinchu, Taiwan). Band intensities were quantified using NIH ImageJ software (1.52v), normalized to loading controls [β-actin (ACTB) or histone H1], and expressed as fold changes relative to control diet-fed *Ppara*^fl/fl^ mice. Each blot was replicated independently at least twice for consistency [37,40]. Full-length blots were demonstrated in Supplementary Information.

### 4.7. Statistical Analysis

Data are presented as mean ± standard error of the means (SEM). Two-way ANOVA was performed to evaluate the effects of genotype (*Ppara*^fl/fl^ vs. *Ppara*^ΔHep^), PEM (control MF diet vs. MF diet containing PEM), and their interaction using GraphPad Prism version 9.5 (GraphPad Software, San Diego, CA, USA). For parameters showing significant interaction, simple effects analysis followed by appropriate pairwise comparisons were conducted. For parameters showing a significant main effect without a significant interaction, pairwise comparisons between groups were performed using Bonferroni correction. A *p*-value < 0.05 was considered statistically significant.

## Data Availability

The original contributions presented in this study are included in the article/Supplementary Material. Further inquiries can be directed to the corresponding author.

## Supplementary Materials

The following supporting information can be downloaded at: https://www.mdpi.com/article/10.3390/ijms27073308/s1. References [41,42] are cited in supplementary document.

## Abbreviations

ACOX1, acyl-CoA oxidase 1; ACTB, β-actin; ALT, alanine aminotransferase; AhR, aryl hydrocarbon receptor; AST, aspartate aminotransferase; AUC, area under the concentration–time curve; BAT, brown adipose tissue; CAR, constitutive androstane receptor; CoA, co-enzyme A; Cmax, maximum plasma concentration; CPT, carnitine palmitoyl-CoA transferase; eWAT, epididymal white adipose tissue; FA, fatty acid; FGF21, fibroblast growth factor 21; Glu, glucose; LACS, long-chain acyl-CoA synthetase; LCAD, long-chain acyl-CoA dehydrogenase; L-FABP, liver fatty acid-binding protein; MASLD, metabolic dysfunction-associated steatotic liver disease; MCAD, medium-chain acyl-CoA dehydrogenase; MTP, microsomal triglyceride transfer protein; NEFA, non-esterified fatty acid; PEM, pemafibrate; PH, peroxisomal hydratase; PL, phospholipid; PPARs, peroxisome proliferator-activated receptors; PPARα, peroxisome proliferator-activated receptor α; PT, peroxisomal thiolase; PXR, pregnane X receptor; qPCR, quantitative polymerase chain reaction; SCAD, short-chain acyl-CoA dehydrogenase; SPPARMα, selective PPARα modulator; TBA, total bile acids; TBARS, thiobarbituric acid reactive substances; T-Chol, total cholesterol; TG, triglyceride; VLCAD, very long chain acyl-CoA dehydrogenase; VLDL, very low-density lipoprotein.
