# Supplementary material for: Hepatocyte PPARα Is Essential for Triglyceride-Lowering Effect of Pemafibrate
Source: Int J Mol Sci. 2026 Apr 6;27(7):3308. doi: 10.3390/ijms27073308 (PMC13072797; doi:10.3390/ijms27073308)

Supporting information

Index of supplementary tables and figure:

Supplementary Table S1. The ingredients of the MF diet.

Supplementary Table S2. Primer pairs used for qPCR analysis.

Supplementary Table S3. Primary antibodies used for western blot analysis.

Supplementary Figure S1. Quantification of serum/liver lipids.

Supplementary Figure S2. Clinically relevant dose of PEM does not affect the expression of genes associated with de novo lipogenesis, TG synthesis, and TG hydrolysis.

Supplementary Figure S3. Histology of the heart and the mRNA expression of FA-oxidizing enzymes in the heart.

Supplementary Figure S4. Histology of the kidney and the mRNA expression of FA-oxidizing enzymes in the kidney.

Supplementary Figure S5. Histology of the BAT and the mRNA expression of FA-oxidizing enzymes in the BAT.

Supplementary Figure S6. Histology of the thigh muscle and the mRNA expression of FA-oxidizing enzymes in the muscle.

Supplementary Figure S7. Histology of the eWAT and the mRNA expression of FA-oxidizing enzymes in the eWAT.

Supplementary Figure S8. The mRNA expression in the eWAT.

Supplementary Figure S9. Immunoblot analysis of PMP70 and catalase in the liver.

Supplementary Figure S10. Hepatic mRNA expression levels of oxidative stress–responsive genes and hepatic TBARS levels.

Supplementary Figure S11. Hepatic mRNA expression levels of target genes of AhR, PXR, and CAR.

Supplementary Figure S12. Full-length blots

Supplementary Table 1. The ingredients of the MF diet (in 100g)

General ingredients		Vitamins		Minerals		Amino acid	
Water (g)	7.9	Vitamin A (IU)	1283	Calcium (g)	1.07	Isoleucine (g)	0.89
Crude protein (g)	23.1	Vitamin D3 (IU)	137	Phosphorus (g)	0.83	Leucine (g)	1.74
Crude fat (g)	5.1	Vitamin E (mg)	9.1	Magnesium (g)	0.24	Lysine (g)	1.24
Crude ash (g)	5.8	Vitamin K3 (mg)	0.04	Sodium (g)	0.19	Methionine (g)	0.44
Crude fiber (g)	2.8	Vitamin B1 (mg)	2.05	Potassium (g)	0.9	Cystine (g)	0.36
Soluble nitrogen-free substance (g)	55.3	Vitamin B2 (mg)	1.1	Iron (mg)	10.6	Phenylalanine (g)	1.04
Calories (kcal)	359	Vitamin C (mg)	4	Aluminum (mg)	2.1	Tyrosine (g)	0.68
		Vitamin B6 (mg)	0.87	Copper (mg)	0.78	Threonine (g)	0.89
		Vitamin B12 (μg)	5.5	Zinc (mg)	4.89	Tryptophan (g)	0.28
		Inositol (mg)	439	Cobalt (mg)	0.1	Valine (g)	1.08
		Biotin (μg)	27	Manganese (mg)	4.84	Arginine (g)	1.42
		Pantothenic acid (mg)	2.45	Ca / P	1.29	Histidine (g)	0.6
		Niacin (mg)	10.61	Ca / Mg	4.5	Alanine (g)	1.2
		Choline (g)	0.18	K / Na	4.77	Aspartic acid (g)	2.14
		Folic acid (mg)	0.17			Glutamic acid (g)	3.99
						Glycine (g)	1.18
						Proline (g)	1.31
						Serin (g)	1.11

MF diet, Maintenance Formula diet.

Supplementary Table 2. Primer pairs used for qPCR analysis.

Gene	Accession #	Primer sequence (5'-3')
18S rRNA	NR_003278	F 5'-CACGGACAGGATTGACAGATTG-3'
		R 5'-CAGACAAATCGCTCCACCAA-3'
Acaa1a	NM_130864	F 5'-TCTACGGTCAACAGACAGTGTTCA-3'
		R 5'-GGCCATGCCAATGTCATAAGA-3'
Acaca	NM_133360	F 5'-GGGCACAGACCGTGGTAGTT-3'
		R 5'-CAGGATCAGCTGGGATACTGAGT-3'
Acadl	NM_007381	F 5'-AGAAACATGGCGGCATTG-3'
		R 5'-CAATATCTGAGTGGAGGCTGAAG-3'
Acadm	NM_007382	F 5'-TGCTTTTGATAGAACCAGACCTACAGT-3'
		R 5'-CTTGGTGCTCCACTAGCAGCTT-3'
Acads	NM_007383	F 5'-CTCCACAGCTAACCTCATCTTTG-3'
		R 5'-GGGTTTGCATGGCTATTTTG-3'
Acadvl	NM_017366	F 5'-GCGTGTGCTCCGAGATATTC-3'
		R 5'-CCAGTGAGTTCCTTTCCTTTG-3'
Acox1	NM_015729	F 5'-TGGTATGGTGTCGTACTTGAATGAC-3'
		R 5'-AATTTCTACCAATCTGGCTGCAC-3'
Acsl1	NM_007981	F 5'-TCCTACGGCAGTGATCTGGTG-3'
		R 5'-GGTTGCCTGTAGTTCCACTTGTG-3'
Adipoq	NM_009605	F 5'-ACTTGTGCAGGTTGGATGGC-3'
		R 5'-AGGACCAAGAAGACCTGCATCT-3'
Apob	NM_009693	F 5'-TCACCCCCGGGATCAAG-3'
		R 5'-TCCAAGGACACAGAGGGCTTT-3'
Cd36	NM_007643	F 5'-CCAAATGAAGATGAGCATAGGACAT-3'
		R 5'-GTTGACCTGCAGTCGTTTTGC-3'
Ces1d	NM_053200	F 5'-TGGTATTTGGTGTCCCATCA-3'
		R 5'-GCTTGGGCGATACTCAAACT-3'
Cidec	NM_178373	F 5'-GCCACGCGGTATTGCCAGGA-3'
		R 5'-GGGTCTCCCGGCTGGGCTTA-3'
Cpt1a	NM_013495	F 5'-TGGCATCATCACTGGTGTGTT-3'
		R 5'-GGTCCGATTGATCTTTGCAATC-3'
Cpt2	NM_009949	F 5'-ATCGTACCCACCATGCACTAC-3'
		R 5'-CTGTCATTCAAGAGAGGCTTCTG-3'
Cyp1a1	NM_009992	F 5'-GGTTAACCATGACCGGGAACT-3'
		R 5'-TGCCCAAACCAAAGAGAGTGA-3'
Cyp1a2	NM_009993	F 5'-GGAGAATGTCACCTCAGGGAAT-3'
		R 5'-CGAAGTTATCATTGAAGGTCTTAAAC-3'
Cyp1b1	NM_009994	F 5'-CCGAAAAGAAAGCGTCTGGG-3'
		R 5'-CCGGGTATCTGGTAAAGAGGATG-3'
Cyp2b10	NM_009999	F 5'-TCCTCAAGTCTTTTATTCAGCTTCG-3'
		R 5'-TGAAGGTTGGCTCAACGACA-3'

Cyp2c55	NM_028089	F 5'-GCTGCCATGGATCCAGTCCT-3'
		R 5'-GGGGCCATAGACTTTTGAGAAATAG-3'
Cyp3a11	NM_007818	F 5'-AGCATTGAGGAGGATCACACAC-3'
		R 5'-TACGAGTCCCATATCGGTAGAG-3'
Cyp3a25	NM_019792	F 5'-CAAATTTCCACATGCCATCA-3'
		R 5'-GCTGCTGGTTACTAGCCTGG-3'
Dgat1	NM_010046	F 5'-CTGCTACGACGAGTTCTTGAGA-3'
		R 5'-GATAGTAGGGACCATCCACTGTTG-3'
Dgat2	NM_026384	F 5'-GCTTCGCGAGTACCTGATGT-3'
		R 5'-CACCACGATGATGATAGCATTG-3'
Dio2	NM_010050	F 5'-TCCTAGATGCCTACAAACAGGTTA-3'
		R 5'-CGGTCTTCTCCGAGGCATAA-3'
Ehhadh	NM_023737	F 5'-CGATACTCTTCCCCCACTACCA-3'
		R 5'-CAGTTACCAACAACGACTCCAATC-3'
Fabp1	NM_017399	F 5'-GCAGAGCCAGGAGAACTTTGAG-3'
		R 5'-TTTGATTTTCTTCCCTTCATGCA-3'
Fasn	NM_007988	F 5'-ATCCTGGAACGAGAACACGATCT-3'
		R 5'-AGAGACGTGTCACTCCTGGACTT-3'
Fgf21	NM_020013	F 5'-CCTCTAGGTTTCTTTGCCAACAG-3'
		R 5'-AAGCTGCAGGCCTCAGGAT-3'
Hmox1	NM_010442	F 5'-TGACACCTGAGGTCAAGCAC-3'
		R 5'-GGCAGTATCTTGCACCAGGC-3'
Lipc	NM_008280	F 5'-ACGGGAAGAACAAGATTGGAAG-3'
		R 5'-CGTTCCCTCAAACATAGGGC-3'
Lipe	NM_010719	F 5'-GAGCGCTGGAGGAGTGTTTT-3'
		R 5'-TGATGCAGAGATTCCCACCTG-3'
Mttp	NM_008642	F 5'-GAGCGGTCTGGATTTACAACG-3'
		R 5'-GTAGGTAGTGACAGATGTGGCTTTTG-3'
Nqo1	NM_008706	F 5'-ACGACAACGGTCCTTTCCAG-3'
		R 5'-GCAGGATGCCACTCTGAATC-3'
Nrf2	NM_010902	F 5'-GGACATGGAGCAAGTTTGGC-3'
		R 5'-CCAGCGAGGAGATCGATGAG-3'
Plin1	NM_175640	F 5'-TGAAGCAGGGCCACTCTC-3'
		R 5'-GACACCACCTGCATGGCT-3'
Pnpla2	NM_025802	F 5'-CGTGTTTCAGACGGAGAGAAC-3'
		R 5'-TTGGAGGGTAGGAGGAATGAG-3'
Scd1	NM_009127	F 5'-AGATCTCCAGTTCTTACACGACCAC-3'
		R 5'-CTTTCATTTCAGGACGGATGTCT-3'
Slc25a20	NM_020520	F 5'-GAGCCGAAACCCATCAGTCC-3'
		R 5'-CAGTCGGACCTTGACCGTG-3'
Slc27a1	NM_011977	F 5'-ACCACCGGGCTTCCTAAGG-3'
		R 5'-CTGTAGGAATGGTGGCCAAAG-3'
Sod1	NM_011434	F 5'-AAGCGGTGAACCAGTTGTGTT-3'
		R 5'-AGCCTTGTGTATTGTCCCCATACT-3'

Sod2	NM_013671	F 5'-TCCCAGACCTGCCTTACGACTAT-3'<br>R 5'-GGTGGCGTTGAGATTGTTCA-3'
Ucp1	NM_009463	F 5'-AGGATGGTGAACCCGACAAC-3'<br>R 5'-GGCCTTCACCTTGGATCTGA-3'

F, forward sequence; R, reverse sequence.
Acaa1a, acetyl-CoA acyltransferase 1A
Acaca, acetyl-CoA carboxylase α
Acadl, long-chain acyl-CoA dehydrogenase
Acadm, medium-chain acyl-CoA dehydrogenase
Acads, short-chain acyl-CoA dehydrogenase
Acadvl, very long-chain acyl-CoA dehydrogenase
Acox1, acyl-CoA oxidase 1
Acsl1, long chain acyl-CoA synthase
Adipoq, C1Q and collagen domain containing
Apob, apolipoprotein B
Cd36, fatty acid translocase
Ces1d, carboxylesterase 1d
Cidec, cell death inducing DFFA like effector c
Cpt1a, carnitine palmitoyl transferase 1a
Cpt2, carnitine palmitoyl transferase 2
Cyp1a1, cytochrome P450, family 1, subfamily a, polypeptide 1
Cyp1a2, cytochrome P450, family 1, subfamily a, polypeptide 2
Cyp1b1, cytochrome P450, family 1, subfamily b, polypeptide 1
Cyp2b10, cytochrome P450, family 2, subfamily b, polypeptide 10
Cyp2c55, cytochrome P450, family 2, subfamily c, polypeptide 55
Cyp3a11, cytochrome P450, family 3, subfamily a, polypeptide 11
Cyp3a25, cytochrome P450, family 3, subfamily a, polypeptide 25
Dgat1, diacylglycerol acyltransferase 1
Dgat2, diacylglycerol acyltransferase 2
Dio2, deiodinase, iodothyronine, type II
Ehhadh, enoyl-Coenzyme A hydratase/3-hydroxyacyl Coenzyme A dehydrogenase
Fabp1, fatty acid binding protein 1
Fasn, fatty acid synthase
Fgf21, fibroblast growth factor 21
Hmox1, heme oxygenase 1
Lipc, hepatic lipase
Lipe, lipase, hormone sensitive
Mttp, microsomal triglyceride transfer protein
Nqo1, NAD(P)H quinone dehydrogenase 1
Nrf2, nuclear factor, erythroid derived 2, like 2
Plin1, perilipin 1
Pnpla2, patatin-like phospholipase domain containing 2

Scd1, stearoyl-Coenzyme A desaturase 1
Slc25a20, solute carrier family 25 (mitochondrial carnitine/acylcarnitine translocase), member 20
Slc27a1, solute carrier family 27 (fatty acid transporter), member 1
Sod1, superoxide dismutase 1
Sod2, superoxide dismutase 2
Ucp1, uncoupling protein 1

Supplementary Table 3. Primary antibodies used for western blot analysis.

Protein name	Abbreviation	Manufacturer	Catalog #	Dilution
Actin, cytoplasmic 1	ACTB	Abcam (Cambridge, MA)	#ab8227	1:1000
Cluster of differentiation 36	CD36	Biotechnology (Dallas, TX)	#sc-9154	1:200
Histone H1	Histone H1	Santa Cruz Biotechnology (Dallas, TX)	#sc-10806	1:200
Microsomal TG transfer protein	MTP	Santa Cruz Biotechnology (Dallas, TX)	#sc-33116	1:200
Peroxisome proliferator-activated receptor α	PPARα	Santa Cruz Biotechnology (Dallas, TX)	#sc-9000	1:200

Other antibodies used were described previously (Aoyama et al., J Biol Chem 1998 and Tanaka et al., Biochem Pharmacol 2010).

Supplementary Figure 1. Quantification of Serum/Liver Lipids.

(A) Serum phospholipid (PL) and total bile acid (TBA) levels.

(B) Liver contents of total cholesterol (T-Chol), triglycerides (TG), non-esterified fatty acid (NEFA), and phospholipid (PL). Data are expressed as the mean ± SEM. ***P < 0.001.

$Ppara^{fl/fl}$, *Ppara*-floxed; $Ppara^{\Delta Hep}$, hepatocyte-specific *Ppara*-disrupted; Con, control MF diet; PEM, MF diet containing 0.00005% pemafibrate. Con n = 6-7, PEM n = 9-10 in each genotype.

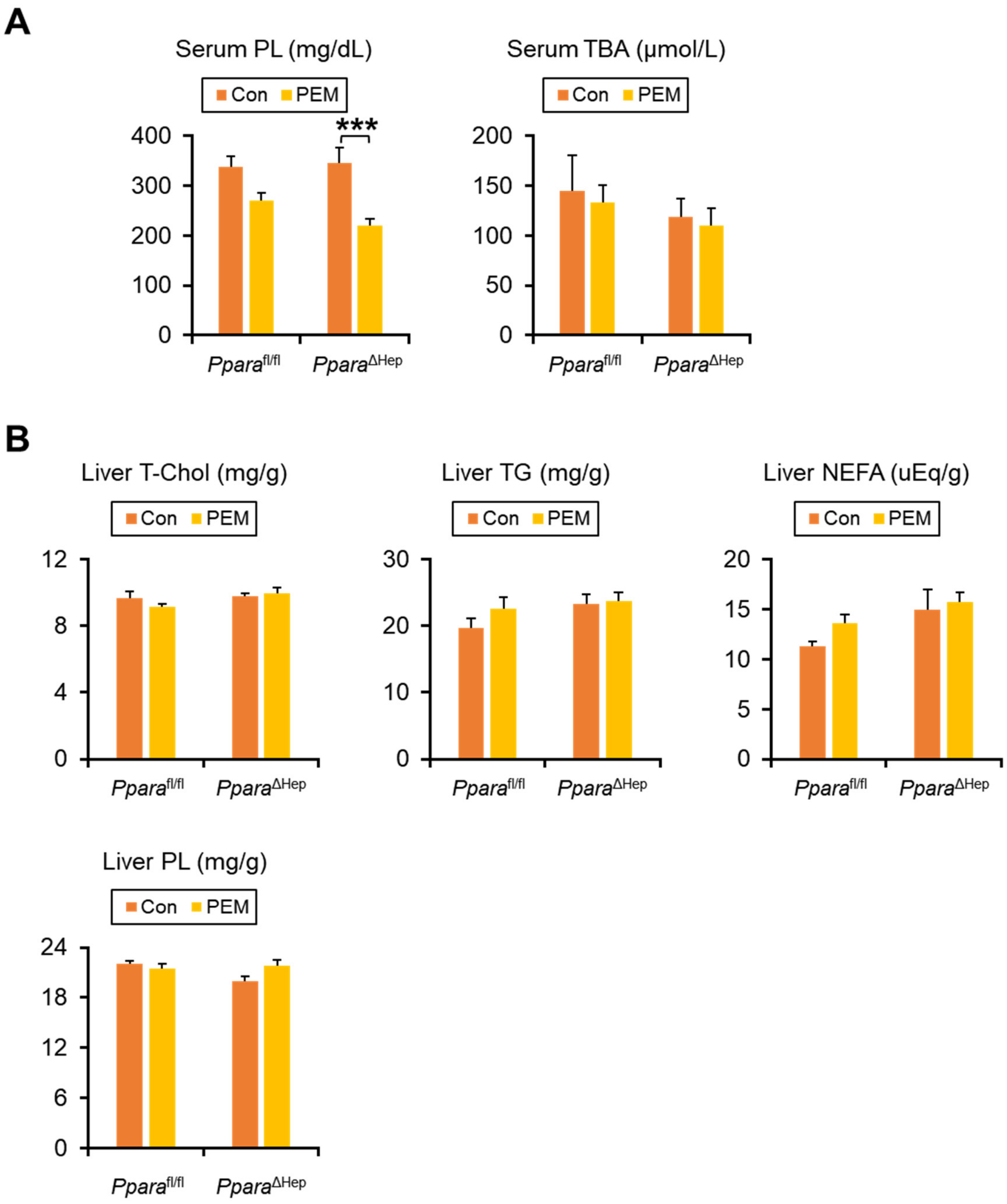
A
Serum PL (mg/dL)
Con PEM
400
300
200
100
0
***
Ppara fl/fl
Ppara ΔHep
Serum TBA (μmol/L)
Con PEM
200
150
100
50
0
Ppara fl/fl
Ppara ΔHep
B
Liver T-Chol (mg/g)
Con PEM
12
8
4
0
Ppara fl/fl
Ppara ΔHep
Liver TG (mg/g)
Con PEM
30
20
10
0
Ppara fl/fl
Ppara ΔHep
Liver NEFA (uEq/g)
Con PEM
20
15
10
5
0
Ppara fl/fl
Ppara ΔHep
Liver PL (mg/g)
Con PEM
24
18
12
6
0
Ppara fl/fl
Ppara ΔHep

Supplementary Figure 2. Clinically Relevant Dose of PEM Does not Affect the Expression of Genes Associated with De Novo Lipogenesis, TG synthesis, and TG hydrolysis.

(A) The mRNA levels of genes encoding *Fasn* and *Acaca* were quantified by qPCR, normalized to that of 18s ribosomal RNA, and expressed as values relative to those of control diet-fed *Ppara*$^{fl/fl}$ mice.

(B) The mRNA levels of genes involved in TG synthesis (*Dgat1*/*2*) and hydrolysis (*Pnpla2* and *Lipc*) were quantified by qPCR, normalized to that of 18s ribosomal RNA, and expressed as values relative to those of control diet-fed *Ppara*$^{fl/fl}$ mice.

Data are expressed as the mean ± SEM. **P* < 0.05, and ***P* < 0.01. *Ppara*$^{fl/fl}$, *Ppara*-floxed; *Ppara*$^{\Delta Hep}$, hepatocyte-specific *Ppara*-disrupted; Con, control MF diet; PEM, MF diet containing 0.00005% pemafibrate. Con n = 6-7, PEM n = 9-10 in each genotype.

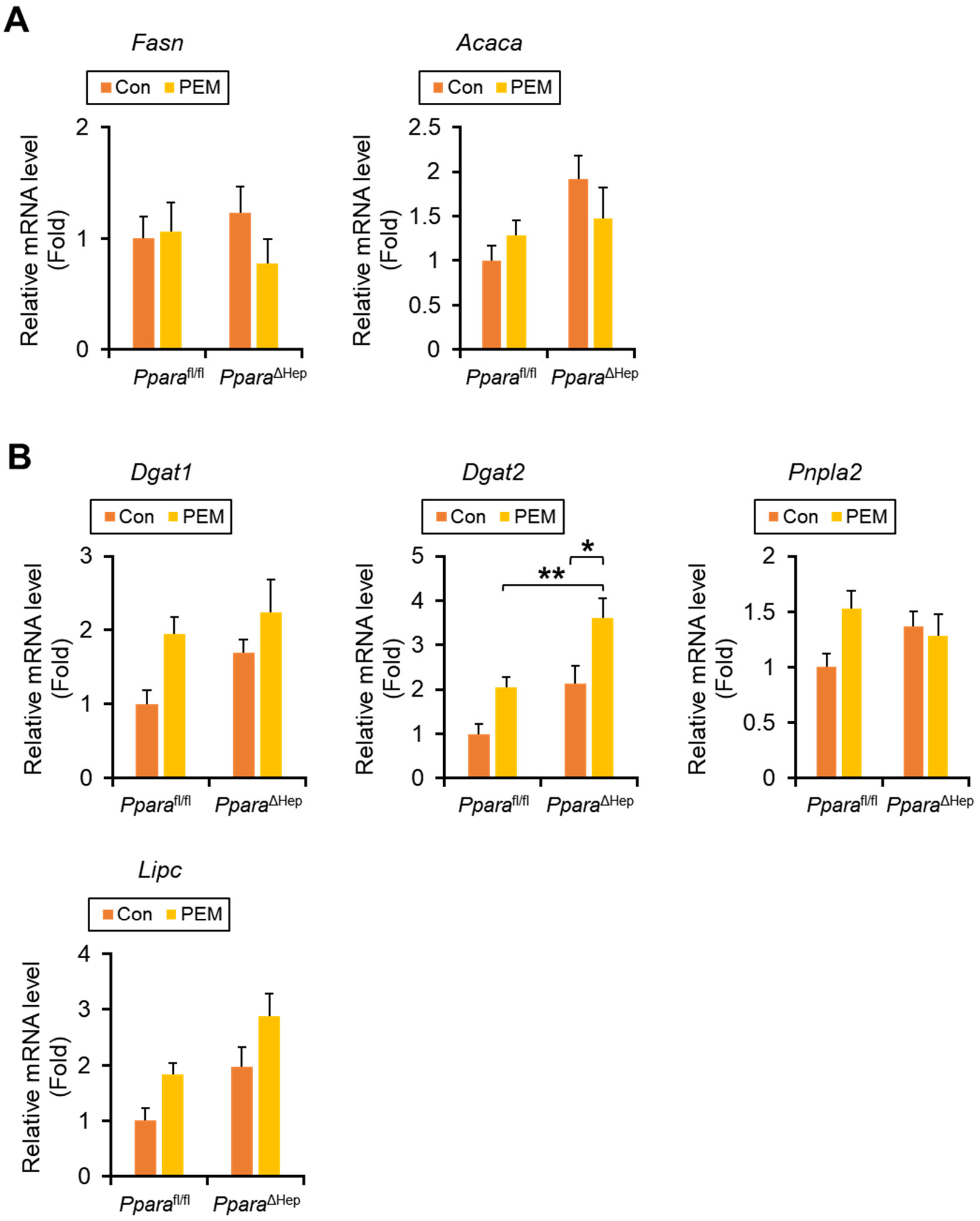

A
Fasn
Con
PEM
Relative mRNA level (Fold)
0
1
2
Ppara fl/fl
Ppara ΔHep
Acaca
Con
PEM
Relative mRNA level (Fold)
0
0.5
1
1.5
2
2.5
Ppara fl/fl
Ppara ΔHep
B
Dgat1
Con
PEM
Relative mRNA level (Fold)
0
1
2
3
Ppara fl/fl
Ppara ΔHep
Dgat2
Con
PEM
Relative mRNA level (Fold)
0
1
2
3
4
5
**
*
Ppara fl/fl
Ppara ΔHep
Pnpla2
Con
PEM
Relative mRNA level (Fold)
0
0.5
1
1.5
2
Ppara fl/fl
Ppara ΔHep
Lipc
Con
PEM
Relative mRNA level (Fold)
0
1
2
3
4
Ppara fl/fl
Ppara ΔHep

Supplementary Figure 3. Histology of the Heart and the mRNA Expression of FA-Oxidizing Enzymes in the Heart.

(A) Representative photomicrographs of hematoxylin and eosin-stained sections of the heart. Scale bar = 50 μm.

(B) The mRNA levels of genes associated with FA-oxidizing enzymes in the peroxisome (*Acox1, Ehhadh,* and *Acaa1a*) and mitochondria (*Acadm* and *Acadl*) were quantified by qPCR, normalized to that of 18s ribosomal RNA, and expressed as values relative to those of control diet-fed *Ppara*$^{fl/fl}$ mice. Data are expressed as the mean ± SEM. **P* < 0.05, and ***P* < 0.01. *Ppara*$^{fl/fl}$, *Ppara*-floxed; *Ppara*$^{\Delta Hep}$, hepatocyte-specific *Ppara*-disrupted; Con, control MF diet; PEM, MF diet containing 0.00005% pemafibrate. Con n = 6-7, PEM n = 9-10 in each genotype.

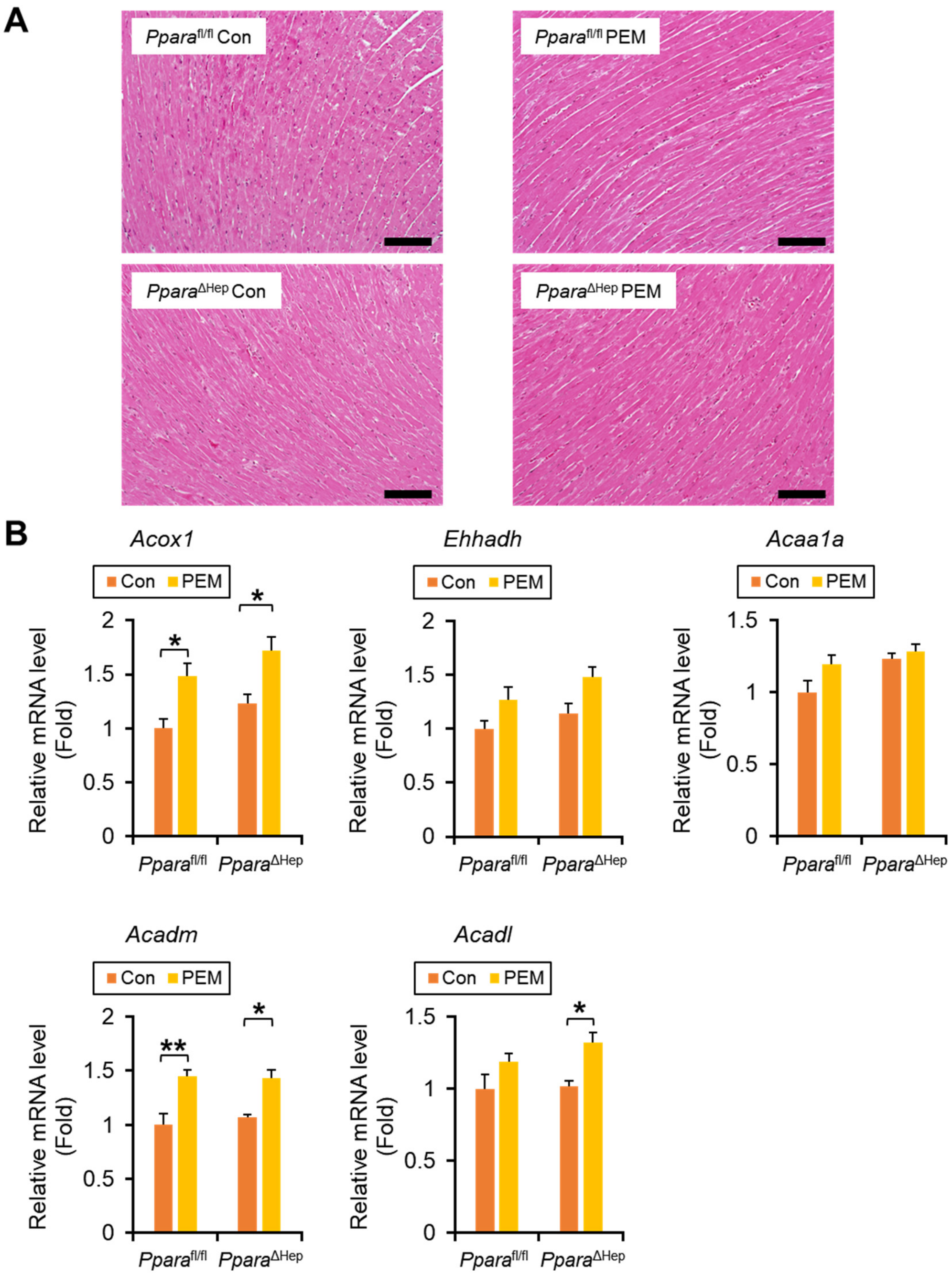
A
Ppara fl/fl Con
Ppara fl/fl PEM
Ppara ΔHep Con
Ppara ΔHep PEM
B
Acox1
Ehhadh
Acaa1a
Acadm
Acadl
Con
PEM
Relative mRNA level (Fold)
Ppara fl/fl
Ppara ΔHep
*
**

Supplementary Figure 4. Histology of the Kidney and the mRNA Expression of FA-Oxidizing Enzymes in the Kidney.

(A) Representative photomicrographs of hematoxylin and eosin-stained sections of the kidney. Scale bar = 50 μm.

(B) The mRNA levels of genes associated with FA-oxidizing enzymes in the peroxisome (*Acox1, Ehhadh,* and *Acaa1a*) and mitochondria (*Acadm* and *Acadl*) were quantified by qPCR, normalized to that of 18s ribosomal RNA, and expressed as values relative to those of control diet-fed *Ppara*$^{fl/fl}$ mice. Data are expressed as the mean ± SEM. *Ppara*$^{fl/fl}$, *Ppara*-floxed; *Ppara*$^{\Delta Hep}$, hepatocyte-specific *Ppara*-disrupted; Con, control MF diet; PEM, MF diet containing 0.00005% pemafibrate. Con n = 6-7, PEM n = 9-10 in each genotype.

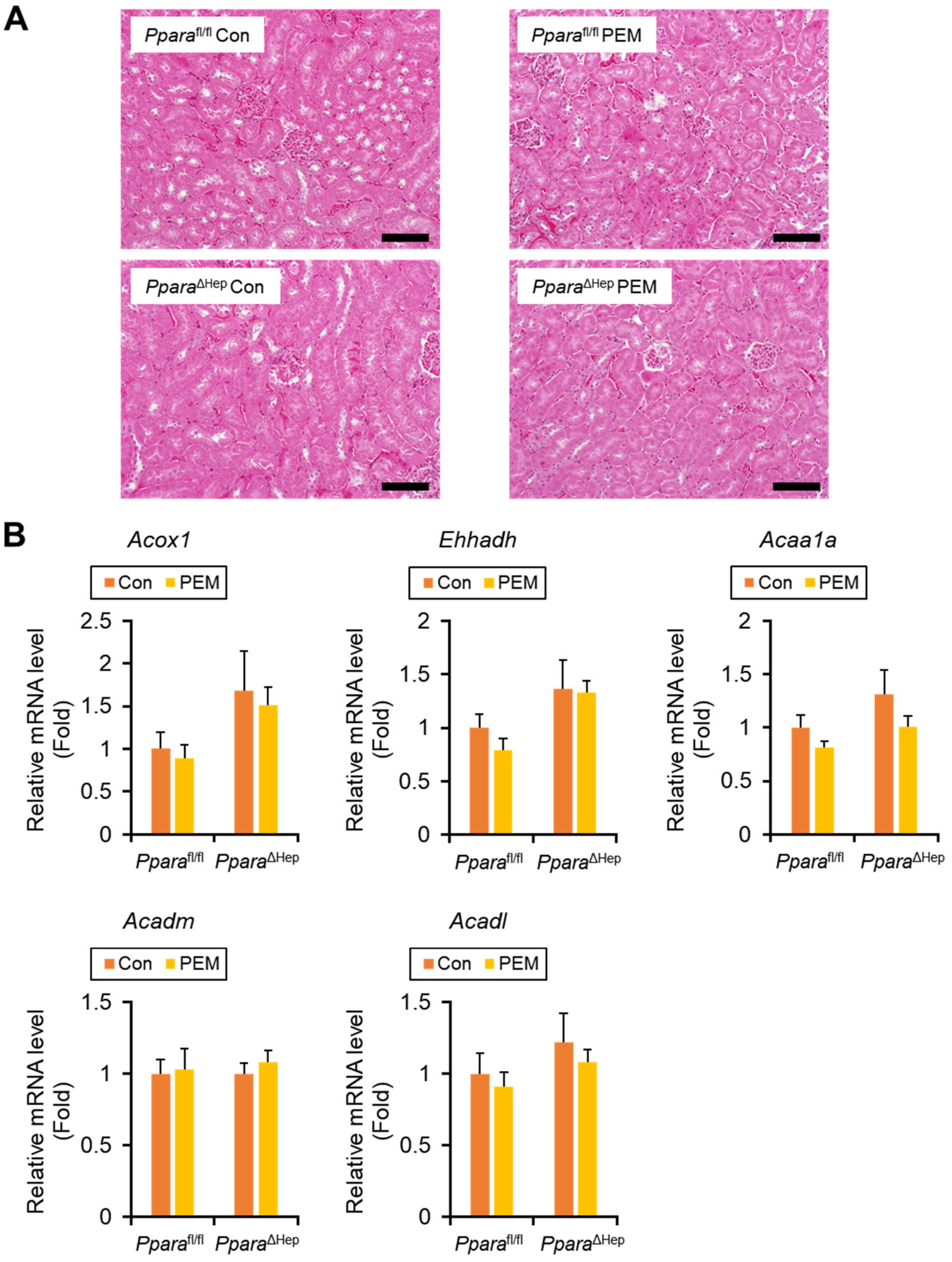

A
Ppara fl/fl Con
Ppara fl/fl PEM
Ppara ΔHep Con
Ppara ΔHep PEM
B
Acox1
Ehhadh
Acaa1a
Acadm
Acadl
Con
PEM
Relative mRNA level (Fold)
2.5
2
1.5
1
0.5
0
Ppara fl/fl
Ppara ΔHep

Supplementary Figure 5. Histology of the BAT and the mRNA Expression of FA-Oxidizing Enzymes in the BAT.

(A) Representative photomicrographs of hematoxylin and eosin-stained sections of the subcapsular BAT. Scale bar = 50 μm.

(B) The mRNA levels of genes associated with FA-oxidizing enzymes in the peroxisome (*Acox1, Ehhadh,* and *Acaa1a*) and mitochondria (*Acadm* and *Acadl*) were quantified by qPCR, normalized to that of 18s ribosomal RNA, and expressed as values relative to those of control diet-fed *Ppara*$^{fl/fl}$ mice. Data are expressed as the mean ± SEM. *Ppara*$^{fl/fl}$, *Ppara*-floxed; *Ppara*$^{\Delta Hep}$, hepatocyte-specific *Ppara*-disrupted; Con, control MF diet; PEM, MF diet containing 0.00005% pemafibrate. Con n = 6-7, PEM n = 9-10 in each genotype.

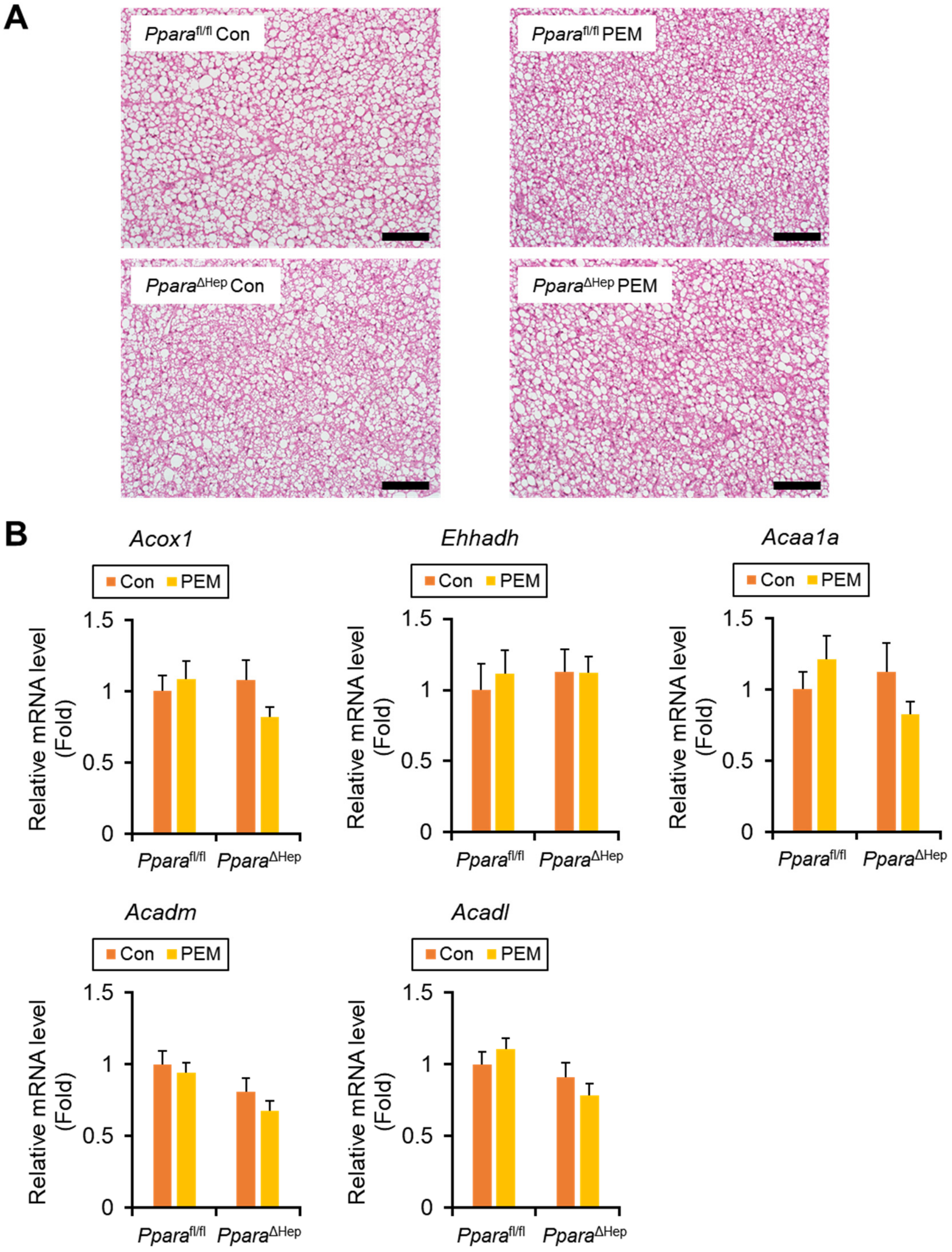
A
Ppara fl/fl Con
Ppara fl/fl PEM
Ppara ΔHep Con
Ppara ΔHep PEM
B
Acox1
Ehhadh
Acaa1a
Acadm
Acadl
Con
PEM
Relative mRNA level (Fold)
1.5
1
0.5
0
Ppara fl/fl
Ppara ΔHep

Supplementary Figure 6. Histology of the Thigh Muscle and the mRNA Expression of FA-Oxidizing Enzymes in the Muscle.

(A) Representative photomicrographs of hematoxylin and eosin-stained sections of the thigh muscle. Scale bar = 50 μm.

(B) The mRNA levels of genes associated with FA-oxidizing enzymes in the peroxisome (*Acox1, Ehhadh,* and *Acaa1a*) and mitochondria (*Acadm* and *Acadl*) were quantified by qPCR, normalized to that of 18s ribosomal RNA, and expressed as values relative to those of control diet-fed *Ppara*$^{fl/fl}$ mice. Data are expressed as the mean ± SEM. *Ppara*$^{fl/fl}$, *Ppara*-floxed; *Ppara*$^{\Delta Hep}$, hepatocyte-specific *Ppara*-disrupted; Con, control MF diet; PEM, MF diet containing 0.00005% pemafibrate. Con n = 6-7, PEM n = 9-10 in each genotype.

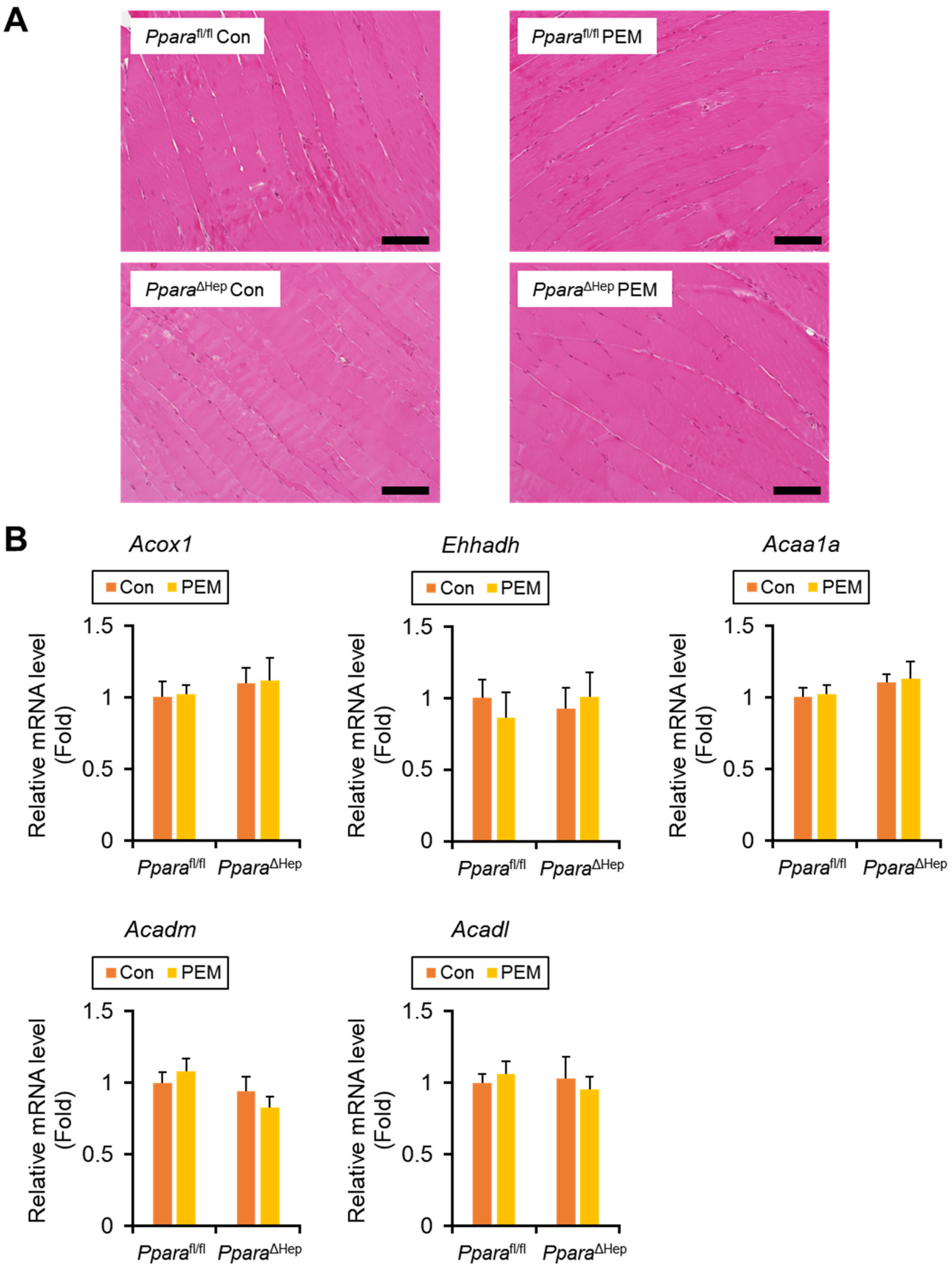
A
Ppara fl/fl Con
Ppara fl/fl PEM
Ppara ΔHep Con
Ppara ΔHep PEM
B
Acox1
Ehhadh
Acaa1a
Acadm
Acadl
Con
PEM
Relative mRNA level (Fold)
1.5
1
0.5
0
Ppara fl/fl
Ppara ΔHep

Supplementary Figure 7. Histology of the eWAT and the mRNA Expression of FA-Oxidizing Enzymes in the eWAT.

(A) Representative photomicrographs of hematoxylin and eosin-stained sections of the eWAT. Scale bar = 50 μm.

(B) The mRNA levels of genes associated with FA-oxidizing enzymes in the peroxisome (*Acox1, Ehhadh,* and *Acaa1a*) and mitochondria (*Acadm* and *Acadl*) were quantified by qPCR, normalized to that of 18s ribosomal RNA, and expressed as values relative to those of control diet-fed *Ppara*$^{fl/fl}$ mice. Data are expressed as the mean ± SEM. *Ppara*$^{fl/fl}$, *Ppara*-floxed; *Ppara*$^{\Delta Hep}$, hepatocyte-specific *Ppara*-disrupted; Con, control MF diet; PEM, MF diet containing 0.00005% pemafibrate. Con n = 6-7, PEM n = 9-10 in each genotype.

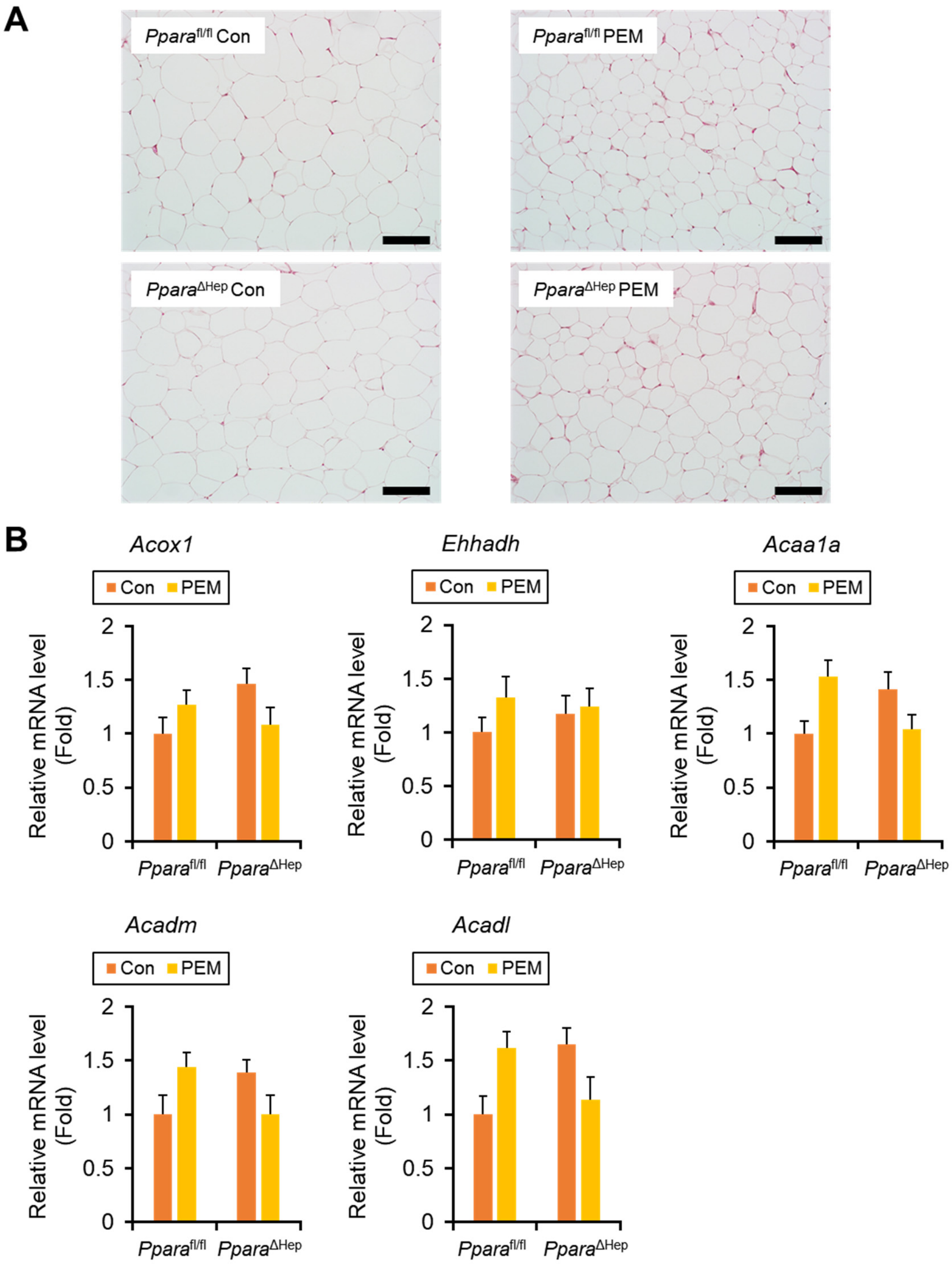
A
Ppara fl/fl Con
Ppara fl/fl PEM
Ppara ΔHep Con
Ppara ΔHep PEM
B
Acox1
Ehhadh
Acaa1a
Acadm
Acadl
Con
PEM
Relative mRNA level (Fold)
0
0.5
1
1.5
2
Ppara fl/fl
Ppara ΔHep

Supplementary Figure 8. The mRNA Expression in the eWAT.

The mRNA levels of genes associated with lipolysis (*Pnpla2*, *Ces1d*, and *Lipe*), lipid droplet stabilization (*Plin1* and *Cidec*), browning (*Dio2* and *Ucp1*) and adipokine secretion (*Adipoq*) were quantified by qPCR, normalized to that of 18s ribosomal RNA, and expressed as values relative to those of control diet-fed *Ppara*$^{fl/fl}$ mice. Data are expressed as the mean ± SEM. $^{**}P < 0.01$. *Ppara*$^{fl/fl}$, *Ppara*-floxed; *Ppara*$^{\Delta Hep}$, hepatocyte-specific *Ppara*-disrupted; Con, control MF diet; PEM, MF diet containing 0.00005% pemafibrate. Con n = 6-7, PEM n = 9-10 in each genotype.

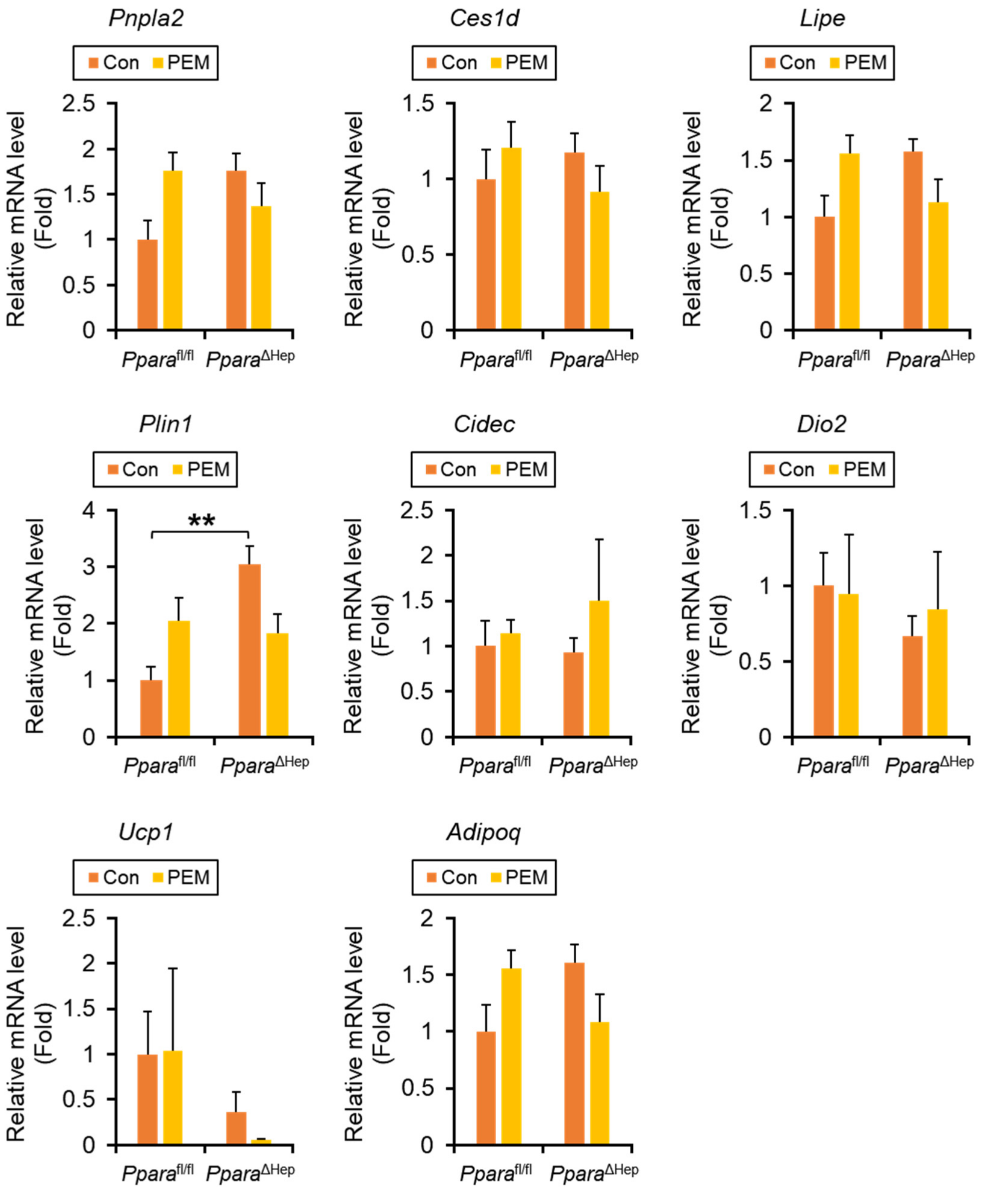

Supplementary Figure 9. Immunoblot analysis of PMP70 and catalase in the liver.

Immunoblot analysis of PMP70 and catalase in the liver. The ACTB was used as a loading control. Band intensity was measured densitometrically, normalized to that of ACTB, and expressed as values relative to those of control diet-fed *Ppara*$^{fl/fl}$ mice. Results were obtained from 2 independent immunoblot experiments. Data are expressed as the mean ± SEM. ***P* < 0.01. *Ppara*$^{fl/fl}$, *Ppara*-floxed; *Ppara*$^{\Delta Hep}$, hepatocyte-specific *Ppara*-disrupted; Con, control MF diet; PEM, MF diet containing 0.00005% pemafibrate. Con n = 6-7, PEM n = 9-10 in each genotype.

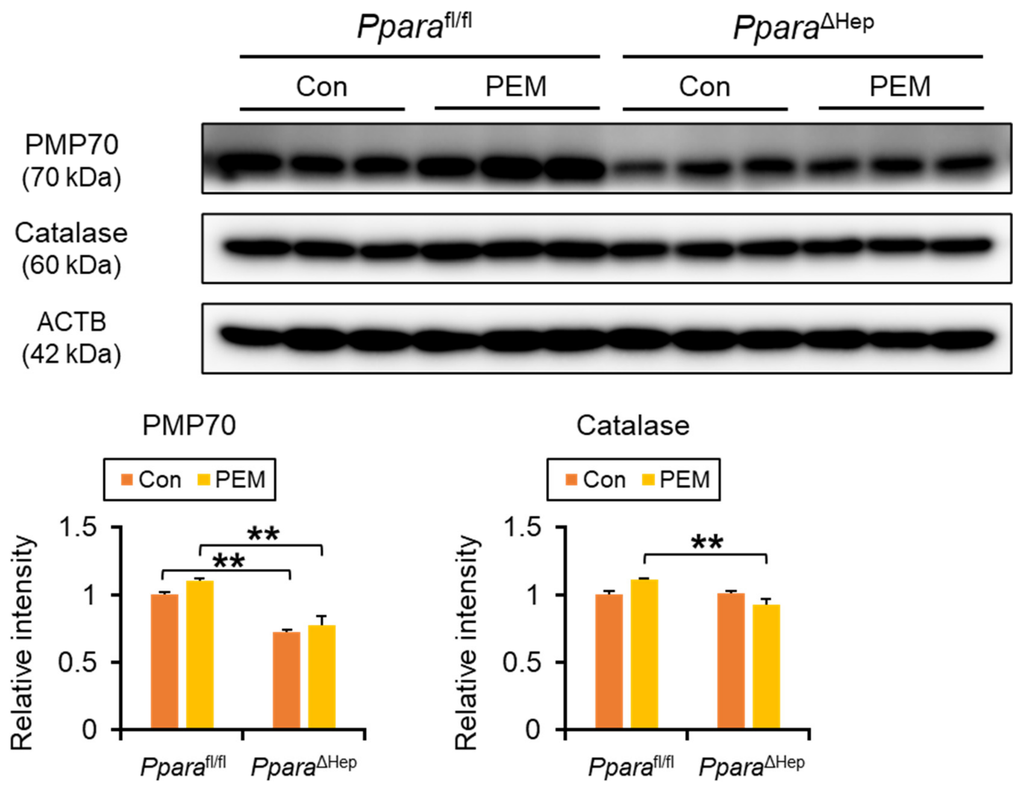

Supplementary Figure 10. Hepatic mRNA expression levels of oxidative stress–responsive genes and hepatic TBARS levels.

(A) Hepatic mRNA expression levels of oxidative stress–responsive genes (*Nrf2*, *Sod1*, *Sod2*, *Hmox1*, and *Nqo1*)

(B) Hepatic TBARS levels.

Data are expressed as the mean ± SEM. *Ppara*$^{fl/fl}$, *Ppara*-floxed; *Ppara*$^{\Delta Hep}$, hepatocyte-specific *Ppara*-disrupted; Con, control MF diet; PEM, MF diet containing 0.00005% pemafibrate. Con n = 6-7, PEM n = 9-10 in each genotype.

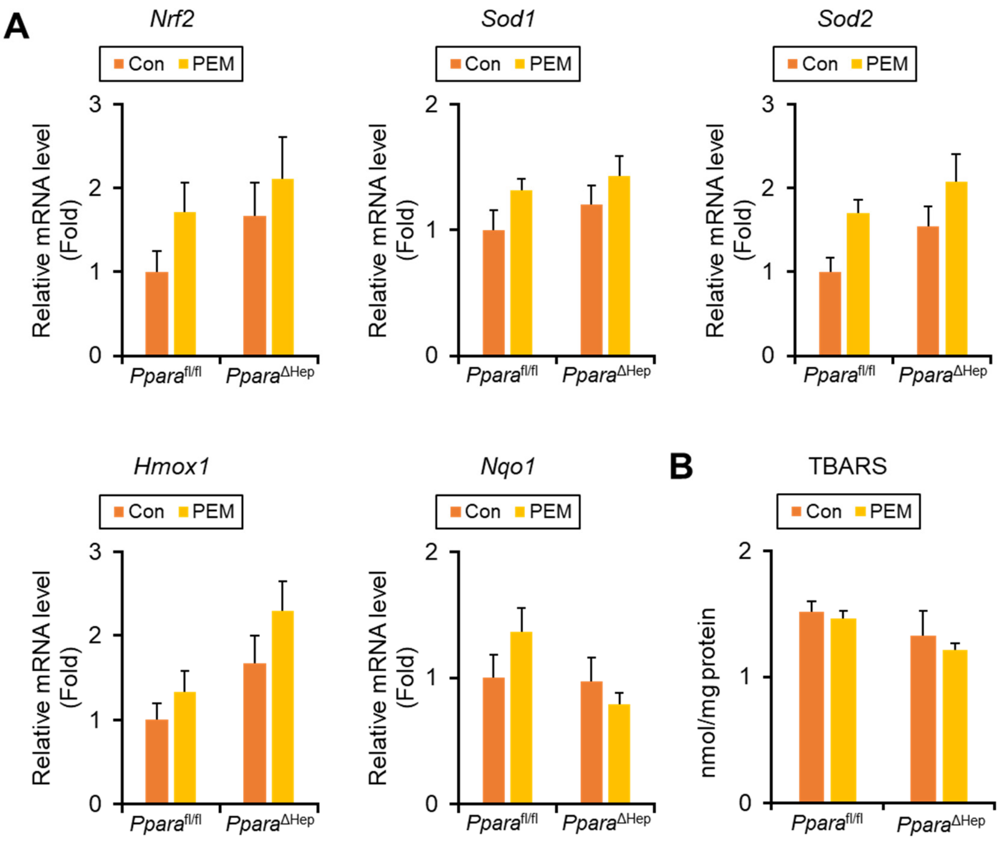

Supplementary Figure 11. Hepatic mRNA expression levels of target genes of AhR, PXR, and CAR.

(A) Hepatic mRNA expression levels of AhR target genes (*Cyp1a1*, *Cyp1a2*, and *Cyp1b1*).

(B) Hepatic mRNA expression levels of PXR target genes (*Cyp3a11* and *Cyp3a25*).

(C) Hepatic mRNA expression levels of CAR target genes (*Cyp2b10* and *Cyp2c55*).

Data are expressed as the mean ± SEM. *Ppara*$^{fl/fl}$, *Ppara*-floxed; *Ppara*$^{\Delta Hep}$, hepatocyte-specific *Ppara*-disrupted; Con, control MF diet; PEM, MF diet containing 0.00005% pemafibrate. Con n = 6-7, PEM n = 9-10 in each genotype.

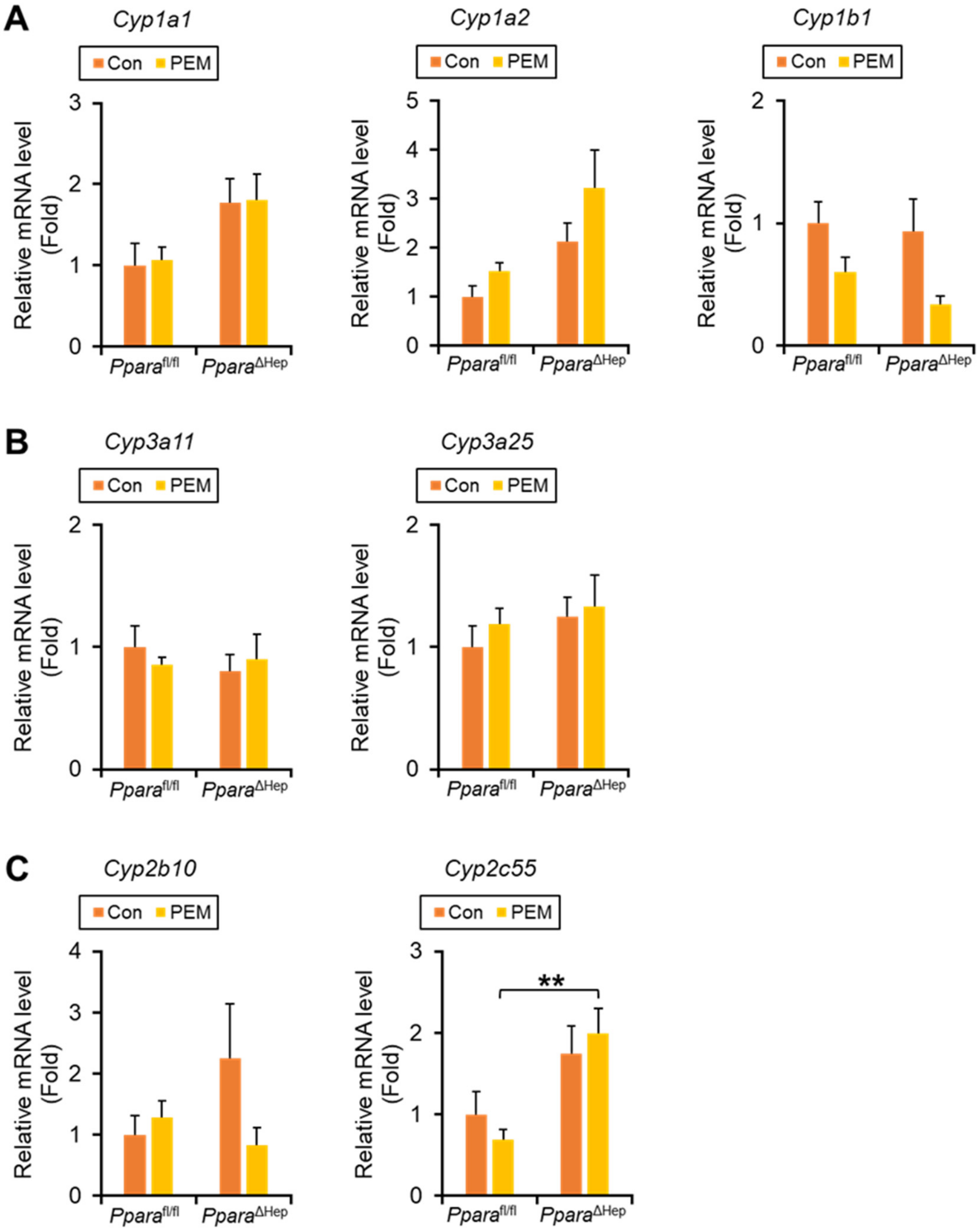
A
Cyp1a1
Con PEM
Relative mRNA level (Fold)
3
2
1
0
Ppara^fl/fl
Ppara^ΔHep
Cyp1a2
Con PEM
Relative mRNA level (Fold)
5
4
3
2
1
0
Ppara^fl/fl
Ppara^ΔHep
Cyp1b1
Con PEM
Relative mRNA level (Fold)
2
1
0
Ppara^fl/fl
Ppara^ΔHep
B
Cyp3a11
Con PEM
Relative mRNA level (Fold)
2
1
0
Ppara^fl/fl
Ppara^ΔHep
Cyp3a25
Con PEM
Relative mRNA level (Fold)
2
1
0
Ppara^fl/fl
Ppara^ΔHep
C
Cyp2b10
Con PEM
Relative mRNA level (Fold)
4
3
2
1
0
Ppara^fl/fl
Ppara^ΔHep
Cyp2c55
Con PEM
**
Relative mRNA level (Fold)
3
2
1
0
Ppara^fl/fl
Ppara^ΔHep

Supplementary Figure 12. Full-length blots.

The protein bands inside the black dotted lines are used.

Figure 1

B

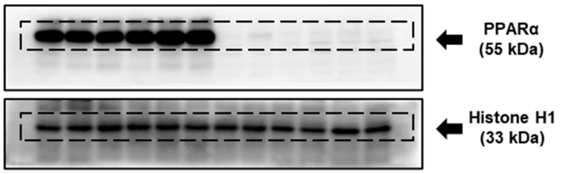

Figure 4

B

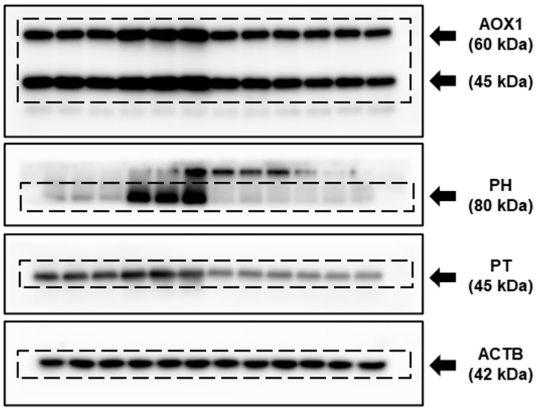

Figure 5

C

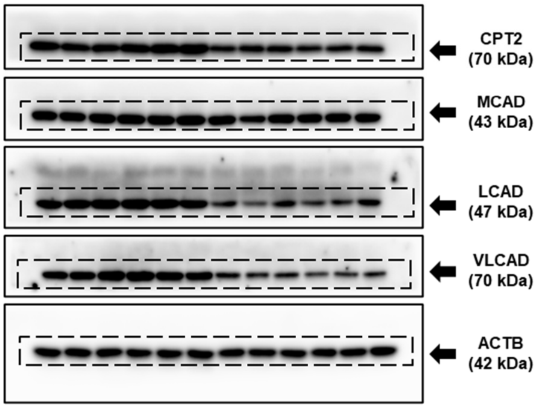

Figure 6

B

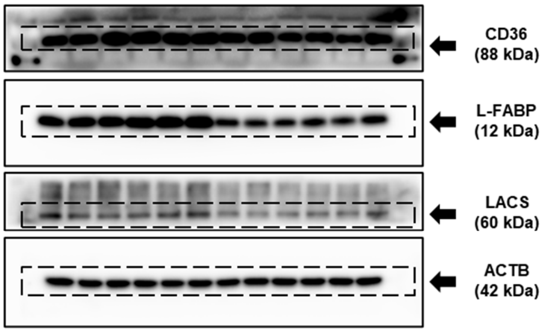

Figure 7

B

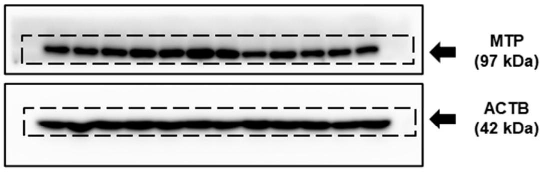

Figure S9

B

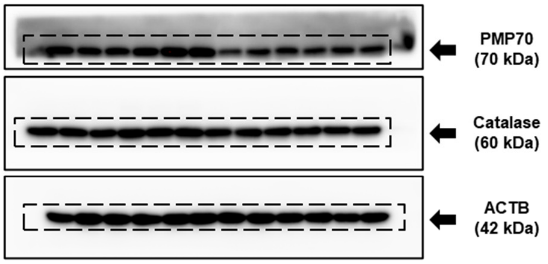

Supplement: Supplementary file 1 [file ijms-27-03308-s001.zip › ijms-4120621-supplementary.pdf]
